# Evolution of structure and spectroscopic properties of a new 1,3-diacetylpyrene polymorph with temperature and pressure

**DOI:** 10.1107/S2052252524003634

**Published:** 2024-05-10

**Authors:** A. Zwolenik, D. Tchoń, A. Makal

**Affiliations:** a Biological and Chemical Research Centre, Faculty of Chemistry University of Warsaw Żwirki i Wigury 101 02-089Warszawa Poland; b Molecular Biophysics and Integrated Bioimaging Division Lawrence Berkeley National Laboratory,1 Cyclotron Road Berkeley CA94720 USA; Sun Yat-Sen University, China

**Keywords:** intermolecular interactions, polymorphism, crystallization and crystal growth, properties of solids, hydrogen bonding, density functional theory, lattice energies, molecular crystals, structure prediction

## Abstract

A new polymorph of 1,3-diacetylpyrene has been obtained from its melt and thoroughly characterized using single-crystal X-ray diffraction, steady-state UV–Vis spectroscopy and periodic density functional theory calculations. Experimental studies covered the temperature range from 90 to 390 K and the pressure range from atmospheric to 4.08 GPa. Optimal sample placement in a diamond anvil cell according to our previously presented methodology ensured over 80% data coverage up to 0.8 Å for a monoclinic sample. Unrestrained Hirshfeld atom refinement of the high-pressure crystal structures was successful and anharmonic behavior of carbonyl oxygen atoms was observed. Unlike the previously characterized polymorph, the structure of 2°AP-β is based on infinite π-stacks of antiparallel 2°AP molecules. 2°AP-β displays piezochromism and piezofluorochromism which are directly related to the variation in interplanar distances within the π-stacking. The importance of weak intermolecular interactions is reflected in the substantial negative thermal expansion coefficient of −55.8 (57) MK^−1^ in the direction of C—H⋯O interactions.

## Abstract 2

A new polymorph of 1,3-diacetylpyrene that is luminescent in the solid state and a prominent negative thermal expansion material has been obtained from its melt. A thorough structural characterization of this new crystal form was performed in a wide temperature and pressure range using single-crystal X-ray diffraction. Structural studies have been combined with steady-state UV–Vis spectroscopy and periodic density functional theory calculations. A previously published methodology of crystal placement in a diamond anvil cell has been successfully applied in predicting optimal 2°AP-β sample orientation, ensuring >80% data coverage and enabling unrestrained Hirshfeld atom refinements for high-pressure structures as well as analysis of anharmonic oscillations.

## 1. Introduction

**Figure 1:**
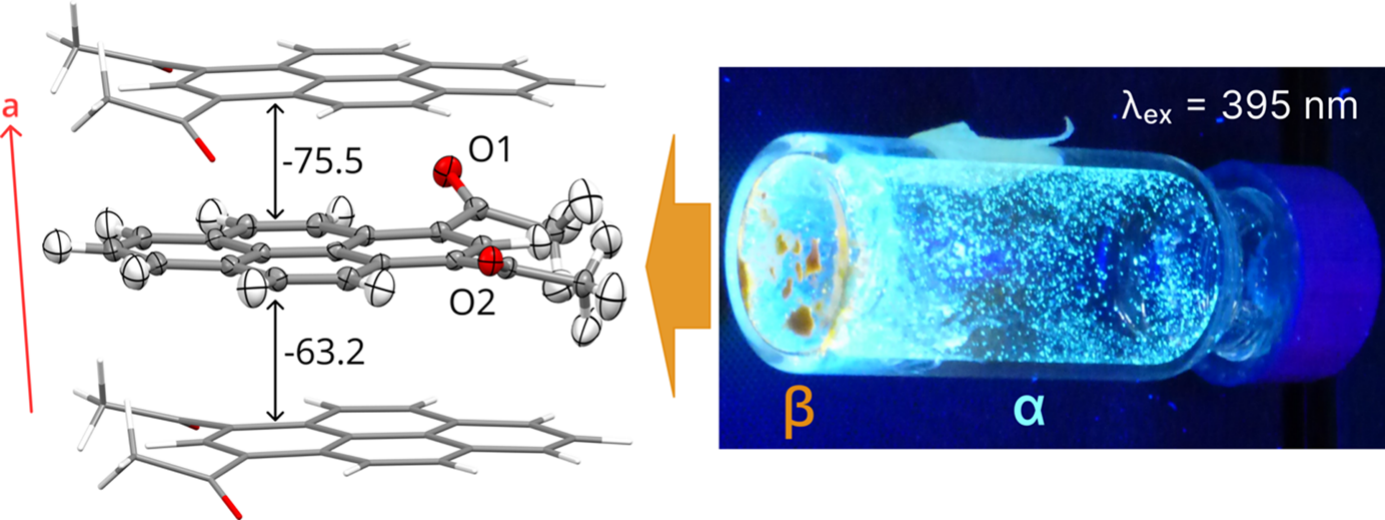
(Left) 2°AP molecule represented with its nearest neighbors within π-stacking, based on the structure of 2°AP-β determined at 100 K. Atomic displacement parameters at the 50% probability level. Intermolecular interaction energies are given in kJ mol^−1^, as estimated by *CrystalExplorer17*. (Right) UV–Vis emissions of 2°AP-α in blue and new 2°AP-β in orange in a vial after the melting experiment. 2°AP-α crystals are the product of resublimation.

With a complex landscape of thermodynamically stable forms and high sensitivity to the environment, materials with organic conjugated systems are a particularly interesting subject for studying structure–property relationships. In this work, we report a new polymorph of 1,3-diacetylpyrene, denoted 2°AP-β, which features distinct π-stacks, unlike previously reported and characterized 2°AP-α (Rajagopal *et al.*, 2014 ▸; Tchoń & Makal, 2021 ▸), leading to significantly red-shifted strong photoluminescence (Fig. 1 ▸), and which we also found to exhibit negative thermal expansion (NTE) over a 300 K temperature range. Application of external stimuli provides means to modify crystal structures in a controllable manner, allowing the study of the intricacies of intermolecular interactions, provided that a very accurate structural model can be proposed.

We decided to employ Hirshfeld atom refinement (HAR) which utilizes aspherical structure factors obtained from single-molecule theoretical calculations to generate tailor-made aspherical form factors for atoms in a molecule and yields very accurate structural information from an X-ray diffraction experiment. A number of studies proved its usefulness, particularly in retrieving neutron-like hydrogen atom positions (Capelli *et al.*, 2014 ▸; Woińska *et al.*, 2016 ▸). HAR has also been successfully applied in a number of cases where various experimental factors negatively affected the X-ray data resolution or interpretability of the Fourier density map, such as modeling of hydrogen atom positions in the vicinity of the heavier elements (Woińska *et al.*, 2023 ▸) or structural studies of metal–organic frameworks (Xu *et al.*, 2023 ▸). However, since the groundbreaking analysis of aromaticity at high pressure conducted by Casati *et al.* (2016 ▸, 2017 ▸), there have been few attempts at application of HAR or quantum crystallographic approaches in general in high-pressure structural analysis. These involved mostly inorganic high-symmetry systems, where challenges inherent to high-pressure structural analysis, such as moderate scattering power, imperfect absorption correction or poor data coverage, have been carefully circumnavigated (Gajda *et al.*, 2020 ▸; Guńka *et al.*, 2021 ▸; Stachowicz *et al.*, 2023 ▸; Chodkiewicz *et al.*, 2022 ▸). Unsurprisingly, structure–property studies of organic materials under increased pressure rely predominantly on geometries obtained from theoretically predicted structures, sometimes without the ability to verify the adequacy of the theoretical model due to a lack of reliable high-pressure experimental geometry (Montisci *et al.*, 2023 ▸).

The monoclinic crystal system and moderate X-ray scattering power of 2°AP-β presented serious challenges for high-pressure structure interpretation. Our contribution is twofold. First, we demonstrate that with the recently proposed approach to improving the quality of high-pressure X-ray diffraction experiments by identifying the most beneficial sample orientation in a diamond anvil cell (DAC) and orienting the sample accordingly (Tchoń & Makal, 2021 ▸), it was possible to collect single-crystal high-pressure diffraction data suitable for interpretation with an unrestricted HAR approach. Second, we show that the resulting structures not only yield theoretical predictions in excellent agreement with spectroscopy experiments, but also allow for experimental confirmation of the presence of significant anharmonic vibrations, which have been linked to NTE phenomenon (Miller *et al.*, 2009 ▸; Attfield, 2018 ▸).

The rest of this paper is structured as follows: Section 2 details the experimental and theoretical methods used, followed by a discussion of the crystal structure at ambient pressure given in Section 3.1, high-pressure structural determinations and multi-temperature structural analysis in Sections 3.2 and 3.3, and the results of UV–Vis spectroscopy in Section 3.4.

## 2. Experimental

### 2.1. Sample preparation

1,3-diacetylpyrene was synthesized using the protocol previously applied to obtain 1,8-diacetylpyrene (Tchoń *et al.*, 2021 ▸). Recrystallization from a range of solvents of varying polarity yielded mainly block crystals of the known α polymorph, with traces of a new monoclinic β phase identified due to its distinct orange fluorescence (Fig. 1 ▸). Specimens of 2°AP-β suitable for single-crystal X-ray diffraction could only be obtained by melting large blocks of 2°AP-α on a heating stage, followed by rapid cooling. The melting point of the new phase has been determined to be 174°C. Detailed procedures are described in Sections S1 and S2 of the supporting information. A single crystal of 2°AP-β was either mounted on a nylon/mitegen loop or a glass capillary with a trace of epoxy resin for the variable-temperature experiments, or placed in a DAC for the variable-pressure experiments.

### 2.2. X-ray diffraction experiments

The following X-ray diffraction experiments were conducted: (1) several series of *in house* measurements for structure or unit-cell determination at 90–390 K, a total of five crystal specimens were used; (2) a series of synchrotron experiments for structure determination at pressures up to 2.0 GPa; (3) a series of low-resolution *in house* experiments for unit-cell determination and systematic extinction analysis at 0.3–1.6 GPa. Details are listed in Section S3. The crystals of 2°AP-β were only moderately diffracting, making *in house* high-pressure structure determination unfeasible.

In order to collect reliable diffraction data at increased pressure and ensure satisfactory data completeness, it was necessary to conduct the experiment with the shortest practicable X-ray wavelength, in a DAC with a high opening angle, and with the sample placed in the most beneficial orientation (Tchoń & Makal, 2021 ▸). To achieve these objectives, a single crystal of 2°AP-β was placed in an Almax DACOne20 DAC with an effective 2θ opening angle of 52°. According to the potency map (Fig. S3.1 of the supporting information) for the monoclinic sample at the given experimental setup, maximal data coverage can be obtained when the main crystallographic directions are not aligned with the DAC axis. For optimal crystal orientation, after pre-experiment and tentative face-indexing, the specimen was propped on the diamond cullet with a drop of poxipol glue (Fig. S3.2) to avoid any (*h*0*l*) as well as (010) crystal faces being parallel to the diamond face. Low-density silicone oil was used as a pressure-transmitting medium (PTM). The X-ray diffraction data were collected at the CRISTAL beamline at the SOLEIL synchrotron (λ = 0.42 Å). Equipped with a six-circle goniometer, this setup enabled us to collect all available diffraction data with redundancy of over 5 up to a resolution of at least 0.7 Å. Diffraction experiments were performed at 0.85, 1.14 and 1.71 GPa. Structural data from higher pressure were unavailable due to beam time limitations and sample deterioration (Fig. S3.3).

*In house* experiments at variable pressure were conducted using a Rigaku Oxford Diffraction Supernova diffractometer equipped with an Mo *K*α X-ray microsource (λ = 0.71 Å). A single crystal of 2°AP-β was placed in a DAC of Merill–Basset design with an effective opening angle of 32° with low-density silicone oil as a PTM. The data were collected in *CrysAlisPro* (Rigaku Oxford Diffraction, 2019 ▸). The pressure in a DAC was determined using the R1 fluorescence line from reference ruby spheres, based on the calibration curve determined by Piermarini *et al.* (1975 ▸) with Ragan’s temperature correction (Ragan *et al.*, 1992 ▸). Experiments at variable temperature were also conducted on a Rigaku Oxford Diffraction Supernova using either an Mo *K*α or a Cu *K*α X-ray microsource. An Oxford Cryosystem 700 was used for temperature control.

Data reduction was performed with *CrysAlisPro* (Rigaku Oxford Diffraction, 2019 ▸). In the case of measurements at high pressure, a numerical multi-scan absorption correction was used, partly correcting for the diamond absorption. For multi-temperature measurements, an analytical absorption correction from the crystal shape was applied.

Structure determination was performed by direct methods [*ShelXS* (Sheldrick, 2008 ▸)] or intrinsic phasing [*ShelXT* (Sheldrick, 2015 ▸)]. All structures were initially refined using the least-squares method as implemented in *ShelXL* (Sheldrick, 2008 ▸). All hydrogen atoms could be located directly from the electron density map in the case of both low-temperature (less than 300 K) and high-pressure structural diffraction experiments. At this stage, all hydrogen atoms were refined in riding approximation (AFIX instructions in *SHELXL*) which restrains C—H distances to prescribed values and the isotropic displacement parameters of hydrogen atoms to that of the C atom multiplied by a constant (Sheldrick, 2015 ▸).

In order to obtain a better description of experimental geometry and electron density distribution, selected structures were further refined using aspherical atomic scattering factors from *MATTS* (Jha *et al.*, 2022 ▸) applied with *DISCaMB* (Chodkiewicz *et al.*, 2018 ▸) within *olex2.refine* (Dolomanov *et al.*, 2009 ▸). At this stage, all hydrogen atoms were refined unrestrained with anisotropic atomic displacement parameters (ADPs) for all but the highest pressure point.

The same subset of structures was finally subject to HAR using *NoSpherA2*, an implementation of non-spherical atomic form-factors in *Olex2* (Kleemiss *et al.*, 2021 ▸). For each structure the electron density was calculated from a Gaussian basis set single-determinant SCF wavefunction for a single molecule in an asymmetric unit in the exact experimental geometry. The effects of temperature/pressure would thus be indirectly manifested in the molecular geometry. Calculations were performed with *ORCA* (version 5.0; Neese *et al.*, 2020 ▸) using the density functional theory (DFT) approach with B3LYP functional, 6-31G(d,p) basis set and Grimme D3 dispersion correction.

All hydrogen atoms were refined independently with anisotropic ADPs for all but the highest pressure point, where similarity restraints (SADI, SIMU) were applied to C—H distances and ADPs of methyl hydrogen atoms and rigid bond restraints (RIGU) were used for aromatic C—H bonds (Sheldrick, 2015 ▸). At 295 K and 0.85 GPa, anharmonic corrections in the Gramm–Charlier approximation (GC) for the oxygen atoms were refined alongside other parameters using *olex2.refine* (Kuhs, 1988 ▸; Volkov *et al.*, 2023 ▸; OlexSys, 2020 ▸). The quality of the final models and applicability of the anharmonic description of atomic displacements were verified by the fractal plots (Meindl & Henn, 2008 ▸) and estimation of minimum necessary data resolution (Kuhs, 1988 ▸). Data collection and refinement statistics are summarized in Table 1 ▸ and in Section S5. Final structures were deposited with the CCDC (CCDC Numbers 2236014–2236020; Table S5.2 of the supporting information).

### 2.3. Luminescence

The steady-state UV–Vis fluorescence spectra were recorded in the pressure range 1 atm to 4.26 GPa with a Labram HR800 (Horiba JobinYvon) spectrometer coupled with an Olympus BX61 confocal microscope and diode-pumped Nd:YAG laser of 405 nm as the excitation source (Section S6 of the supporting information). The spectra were collected for the same single-crystal specimen in a DAC which was used for *in house* high-pressure X-ray diffraction experiments.

### 2.4. Periodic DFT calculations

In order to retrieve structural and electron density characteristics of 2°AP-β in the whole spectroscopically investigated pressure range, the experimental crystal geometry from 100 K was fully optimized in *Crystal17* (Dovesi *et al.*, 2018 ▸). Equation of state calculations were also performed, starting from the experimental geometry. Periodic DFT calculations were performed with B3LYP functional, 6-311G basis-set and Grimme D3 dispersion correction. The lattice energy, π-stacking interaction energies and band-gap spans were obtained. Details are summarized in Section S7 of the supporting information.

### 2.5. Other approaches

Estimation of compressibility and expansion moduli based on experimental and theoretical unit-cell parameters was performed using the *PASCal* (Cliffe & Goodwin, 2012 ▸) server (2022 version). Calculations of approximate intermolecular energies were also performed for high-quality structures resulting from refinements with aspherical atomic scattering factors as well as theoretically predicted high-pressure structures using *CrystalExplorer* (Mackenzie *et al.*, 2017 ▸) with the DFT method [B3LYP/6-31G(d,p)].

## 3. Results and discussion

### 3.1. Crystal structure of 2°AP-β at ambient pressure

2°AP in its β form crystallizes in the monoclinic system in space group *P*2_1_/*c* with one molecule in the asymmetric unit. The geometries of single molecules are similar in both polymorphs, with carbonyl oxygen atoms tilted in the same direction out of the pyrene plane. The main difference is the absence of intramolecular *m*-plane symmetry in the case of 2°AP-β. As a result, two carbonyl moieties in 2°AP-β are tilted out of the pyrene plane by two distinct angles, O1 by only 17° and O2 by 24° (the tilt in the case of 2°AP-α was over 32°). This difference is less pronounced at room temperature but becomes more prominent with increased pressure (Table S8.2).

2°AP molecules form distinct π-stacks along the [100] crystallographic direction (Fig. 1 ▸). According to both periodic DFT calculations and *CrystalExplorer17* estimates, these are the strongest intermolecular interactions (detailed tables in Section S10 of the supporting information), with energies exceeding −70 kJ mol^−1^ between molecules whose carbonyl oxygen atoms are directed towards each other. As the other interactions within stacks are less stabilizing by 10 kJ mol^−1^, it can be concluded that the molecules in the stack form dimers analogous to the arrangement observed previously in pyrene aldehyde (Tchoń & Makal, 2019 ▸).

**Figure 2:**
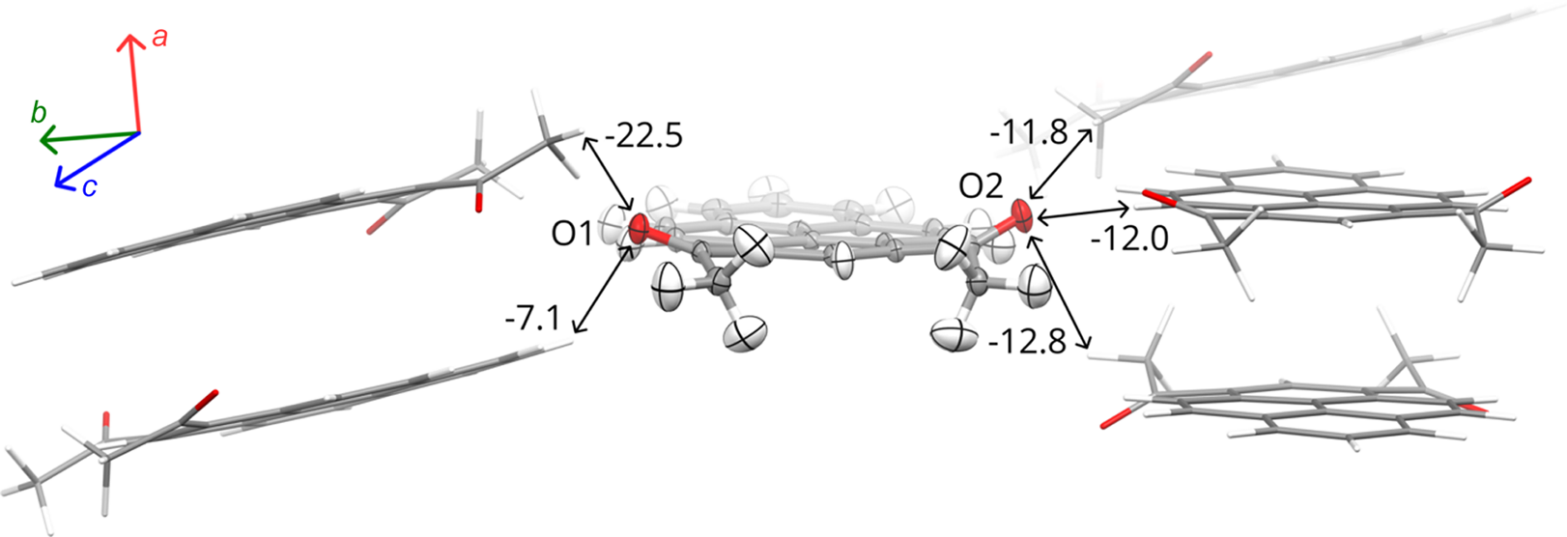
Intermolecular C—H⋯O interactions between the π-stacked columns based on the structure determined at 100 K. Atomic displacement parameters at the 50% probability level. Intermolecular interaction energies are given in kJ mol^−1^, as estimated by *CrystalExplorer17*.

Adjacent stacks viewed along [100] are almost perpendicular and connected by C—H⋯O interactions (Figs. 2 ▸ and S8.8). The strongest, involving O1 oxygen, have interaction energies of over −20 kJ mol^−1^ (Section S10), 2 times stronger than interactions involving O2. They occur between the stacks approximately along the crystallographic [101] direction.

The residual density features at both oxygen positions indicate a possible dynamical disorder above 190 K. At room temperature, these features amount to over 0.5 eÅ^−3^, and with the application of aspherical atomic scattering factors (HAR approach), they allowed the refinement of anharmonic corrections to the ADPs in GC approximation, leading to very satisfactory model statistics (Table 1 ▸).

### 3.2. Reliability of HAR refinements against high-pressure data

Crystal structure analysis under high-pressure is particularly challenging for purely organic molecular crystals, such as 2°AP-β. Two major reasons for this are (1) the moderate to poor X-ray scattering power of such materials, further diminished by placement in a DAC and X-ray absorption by diamonds; and (2) low-symmetry, resulting in poor data coverage which can seriously bias the resulting crystal structure, causing *e.g.* systematic shortening of certain bond lengths. In addition, organic crystals are particularly prone to plastic deformations under pressure, causing quick deterioration of diffracted intensities.

Despite such drawbacks, controlled single-crystal placement in a DAC ensured a potency (Tchoń & Makal, 2021 ▸) of 93% (Fig. S3.1) and actual data completeness of 82–90% for our high-pressure diffraction experiments up to reasonably high resolution (Table 1 ▸).

Structure refinements using aspherical atomic scattering factors in both the databank and the HAR approaches proved to be fully justified. In all instances, *R* factors were notably improved after introducing aspherical atomic scattering factors. Residual density features also became less pronounced and were distributed more randomly, as shown by fractal dimension plots (Fig. S4.1).

The experimental geometries from HAR refinement are in excellent agreement with the results of structure optimization by periodic DFT at a given pressures (*CRYSTAL17*), with an RMS of 0.06 Å or less (Table S7.2).

**Figure 3:**
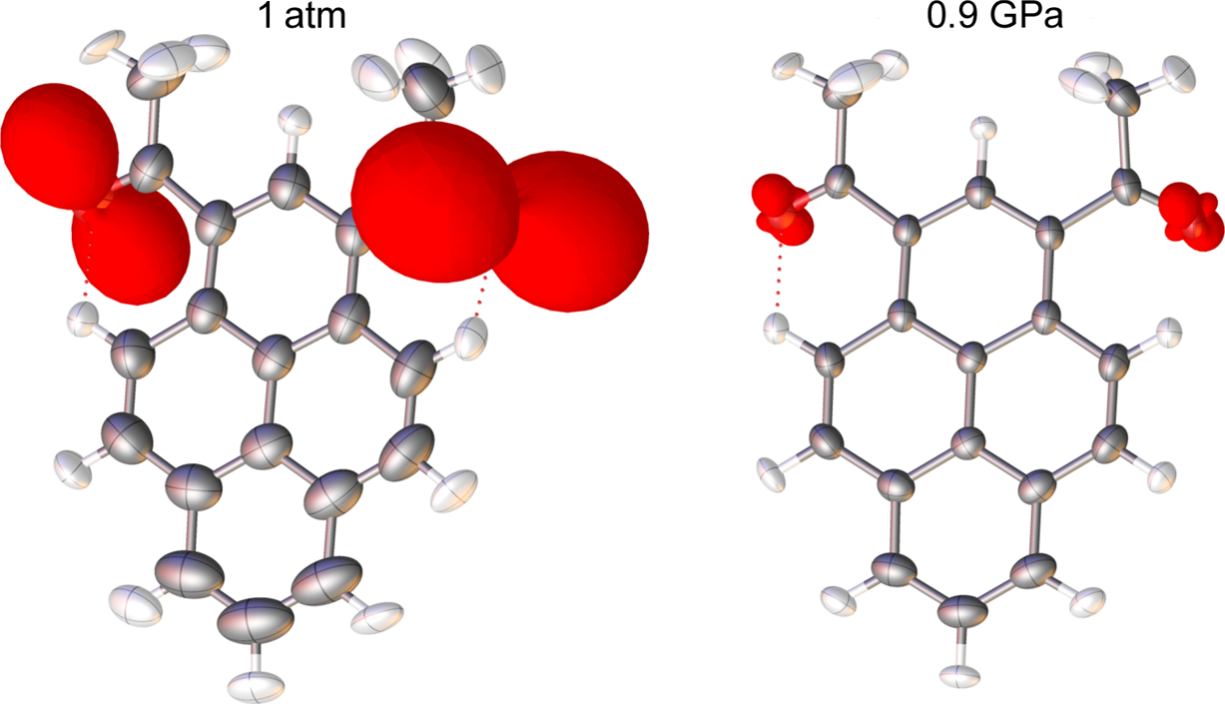
Molecule of HAR-refined 2°AP at 293 K at atmospheric pressure (left) and at 0.85 GPa (right). Harmonic ADPs at the 50% probability level. Anharmonic contributions to the total probability distribution function for the O1 and O2 oxygen atoms are represented as red isosurfaces (peanut-shaped) and scaled by 1.5 for better visibility. Their long axes indicate the direction of anharmonic oscillation of each atom.

In addition to the anisotropic description for all atoms, it was also possible to describe the pronounced out-of-plane motions of the carbonyl moieties in anharmonic approximation, obtaining convergent refinements and meaningful third-order GC parameters at 0.85 GPa (Fig. 3 ▸). We observed a decrease in the extent of anharmonicity between the structures determined at 1 atm and 0.85 GPa. Clearly, our high-pressure data were good enough to be sensitive to this effect.

**Figure 4:**
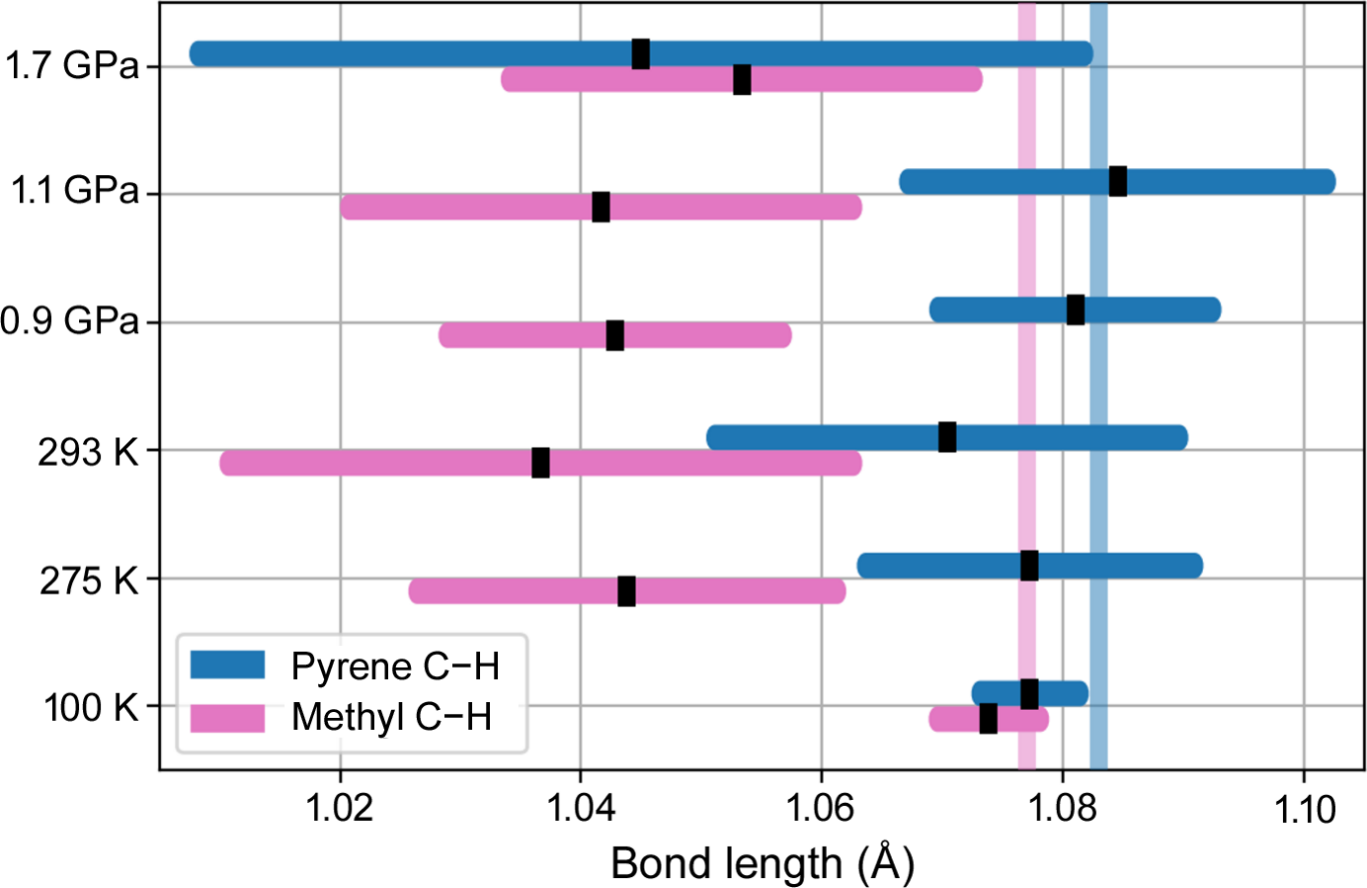
Average C—H bond lengths in pyrene (blue) and methyl (pink) moieties determined by HAR refinements against selected datasets. Theoretical neutron distances were drawn as vertical lines for comparison (Allen & Bruno, 2010 ▸).

The widely discussed reproducibility of the neutron-derived C—H distances (Capelli *et al.*, 2014 ▸; Woińska *et al.*, 2016 ▸; Guńka *et al.*, 2021 ▸) is another measure of the reliability of our high-pressure data. In the case of C—H distances of the pyrene moiety, they remain within the ballpark of the average neutron data for all analyzed datasets with the exception of that at 1.71 GPa (Fig. 4 ▸). The apparent shortening of the C—H distances in the methyl groups at higher temperatures can be perceived as a direct result of the increased librations occurring in these moieties (Goeta & Howard, 2004 ▸; Fucke & Steed, 2010 ▸). Such shortened C—H distances are in agreement with the reference values in the former edition of the *International Tables for Crystallography* (Prince, 2004 ▸) uncorrected for the libration effects (Allen & Bruno, 2010 ▸).

The data collected at 1.71 GPa were already impacted due to crystal deterioration, as illustrated by diminished diffracted intensities and broadening of the diffraction spots (Fig. S3.2). Notably, despite these effects on the dataset, HAR could still be applied with only minor restraints, improving the general model quality.

### 3.3. Structural changes with temperature and pressure

This section summarizes the observations based on all experimental and theoretical data.

#### 3.3.1. Unit-cell constants and bulk properties of 2°AP-β

**Figure 5:**
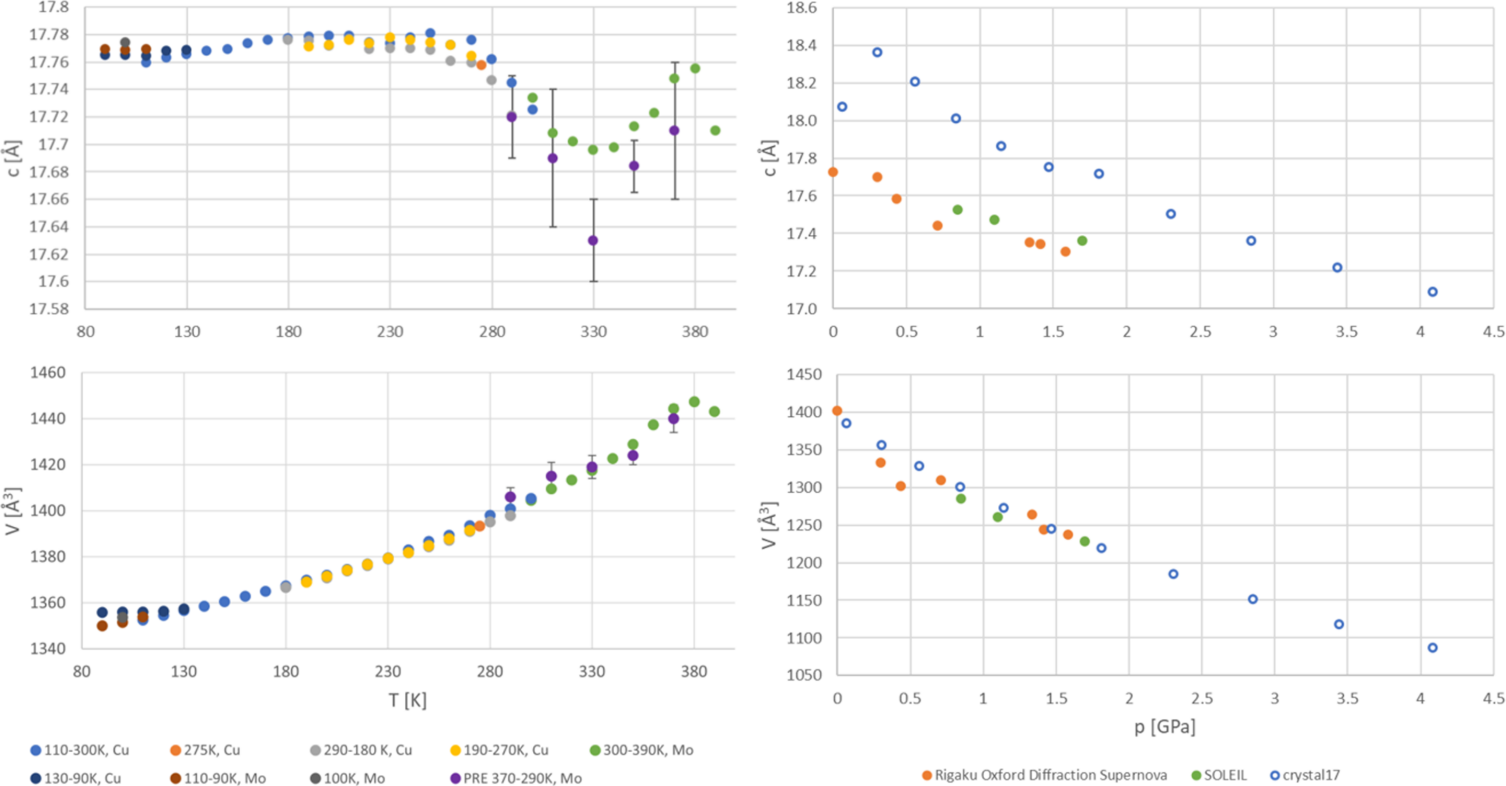
Evolution of the selected unit-cell parameters with external stimuli: temperature (left) and pressure (right), color-coded by experimental series. Purple points, marked as PRE, were obtained from very short control pre-experiments (<30 reflections per pre-experiment), hence the substantial uncertainties. Hollow points indicate the results of the periodic DFT calculations.

The evolution of the crystal structure of 2°AP-β has been investigated for a temperature range from 90 to 390 K at atmospheric pressure and for a pressure range from atmospheric to 1.71 GPa (4.08 GPa theoretically) at room temperature. The unit-cell parameters obtained are summarized in Section S3 of the supporting information and are plotted in Fig. 5 ▸.

The results from different experimental series are very consistent, except for the range from 90 to 130 K, where scattered results indicate that, at lower temperatures, the sample structure is slightly affected by the cooling rate.

The results of the theoretical calculations reproduce the trends in the lattice constants’ changes with pressure and are in excellent agreement with experimentally observed unit-cell volumes. The most inconsistent results were obtained for the *c* parameter, where the experimental values are systematically lower than the theoretically predicted ones. This suggests that the theoretical approach may underestimate the strength of intermolecular interactions in this particular direction.

No phase transitions were induced by either temperature or pressure variation. However, anomalous behavior was noted above 210 K for the unit-cell constant *c*, as it decreases significantly with increased temperature. Contrary to recently summarized tendencies (Kaźmierczak *et al.*, 2021 ▸), the trend for the unit-cell volume expansion with temperature is exponential rather than linear.

**Figure 6:**
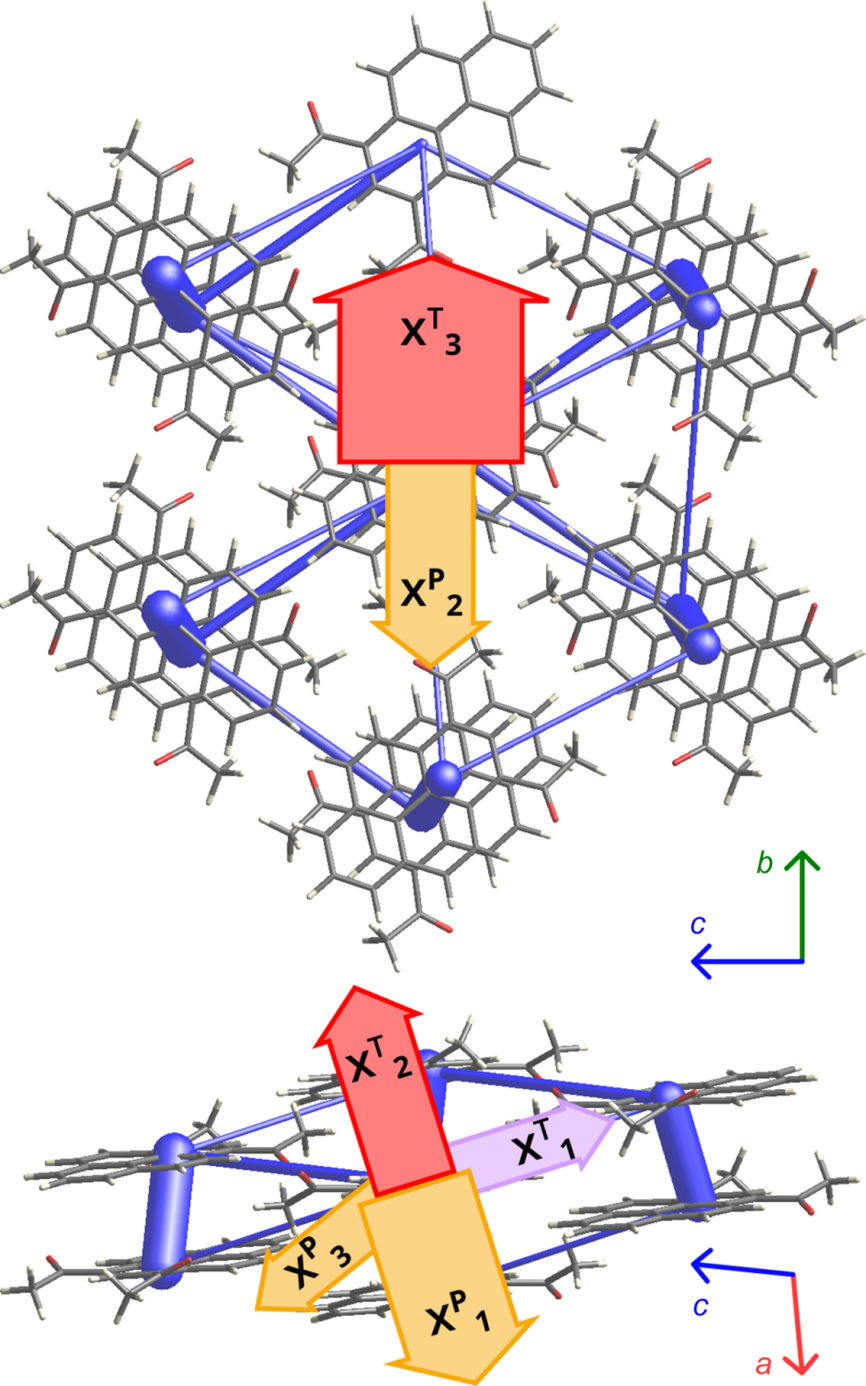
Principal axes of the thermal expansion (red – positive / lavender – negative) and compressibility tensor (orange) superimposed on the intermolecular interaction network in 2°AP-β as calculated using *CrystalExplorer17* for the structure at room temperature. The arrow widths are scaled by the thermal expansion/compressibility coefficients according to Tables S9.14–S9.15.

The principal directions of the thermal expansion and compressibility tensors, estimated using the *PASCaL* server, are very similar, aligning approximately to the [100], [010] and [101] crystallographic directions (Fig. 6 ▸ and Tables S9.1 and S9.2).

The anomalous thermal behavior of 2°AP-β is confirmed by a substantial NTE coefficient of −55.8 (57) MK^−1^ along the [101] crystallographic direction, which coincides with the C—H⋯O interactions. This expansion coefficient is over an order of magnitude more prominent than that observed for graphene (Mann *et al.*, 2017 ▸) and comparable with well know inorganic crystalline NTE materials such as Cu-SIP-3·3H_2_O or BiNiO_3_ perovskite (Cliffe & Goodwin, 2012 ▸; Attfield, 2018 ▸).

Notably, we observe the minimum compressibility of the material in the same direction. This points to the importance of all C—H⋯O interactions in the stabilization of the 2°AP-β structure. Maximum thermal expansion occurs in the direction of the weakest intermolecular interactions ([010], Fig. 6 ▸). Maximum compressibility occurs approximately in the direction of π-stacking ([100] crystallographic direction), which aligns exactly with the vector linking centroids of molecules in inter-dimer π-stacking interaction (Fig. 6 ▸).

The bulk modulus of 2°AP-β, estimated by *PASCal* from the experimental data, is 13.6 (1.6) GPa, a typical value for similar organic systems (Tchoń & Makal, 2019 ▸; Bergantin *et al.*, 2014 ▸).

#### 3.3.2. Evolution of crystal structures and intermolecular interactions

The molecular structure of 2°AP does not change significantly with either temperature or pressure. The only noticeable variation is the increase in the tilting of the oxygen atoms further out of the pyrene plane, O1 by 5% and O2 by over 20%, in the investigated pressure range. However, systematic changes occur in the intermolecular interactions.

**Figure 7:**
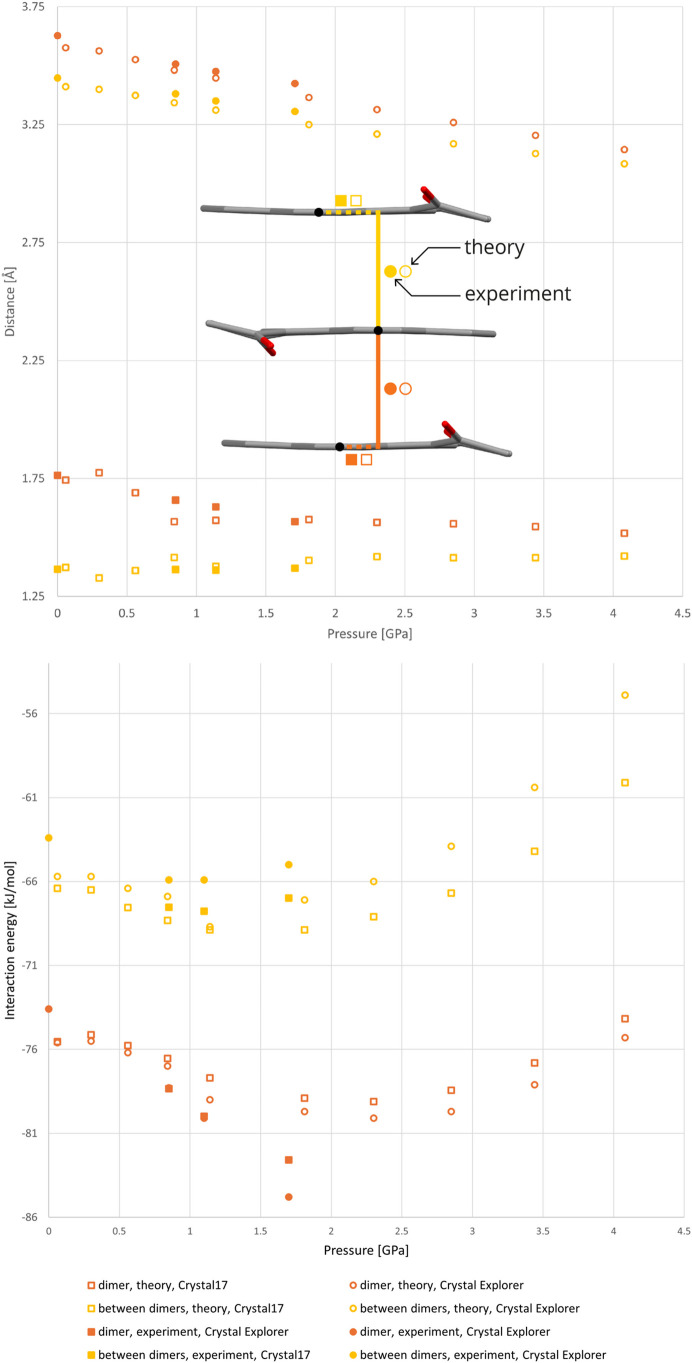
Changes in the energy and geometry of π-stacking-type interactions occurring in the 2°AP-β structure with increasing pressure. The interactions become less stabilizing above 2.00 GPa.

The distances between molecules within the π-stacking and the relative lateral displacements of the pyrene centroids are collected in Table S8.1 and visualized in Fig. 7 ▸. Following a notable compressibility in the π-stacking direction, the intermolecular distances in the stack decrease, maintaining the difference between intra- and inter-dimer interactions. In the experimentally explored pressure range up to 1.71 GPa, molecules forming the dimer shift laterally closer to each other, while the molecules from neighboring dimers maintain their relative lateral positions. Above 1.71 GPa the lateral shifts of the π-stacked molecules are practically constant and their total interaction energies become less negative with increased pressure. Notably, the destabilization is much more pronounced for the interactions between the dimers. A detailed description of intermolecular energies is presented in Table S10.1.

In the case of C—H⋯O interactions, their energies approximated with *CrystalExplorer17* are presented in Tables S10.2–S10.6. The estimates differ significantly between the experimental geometries and those predicted with periodic DFT calculations. For the latter, C—H⋯O appear repulsive above 4.08 GPa. This effect indicates that the structure of 2°AP-β becomes unstable at a relatively low pressure.

### 3.4. Optical properties of the material

**Figure 8:**
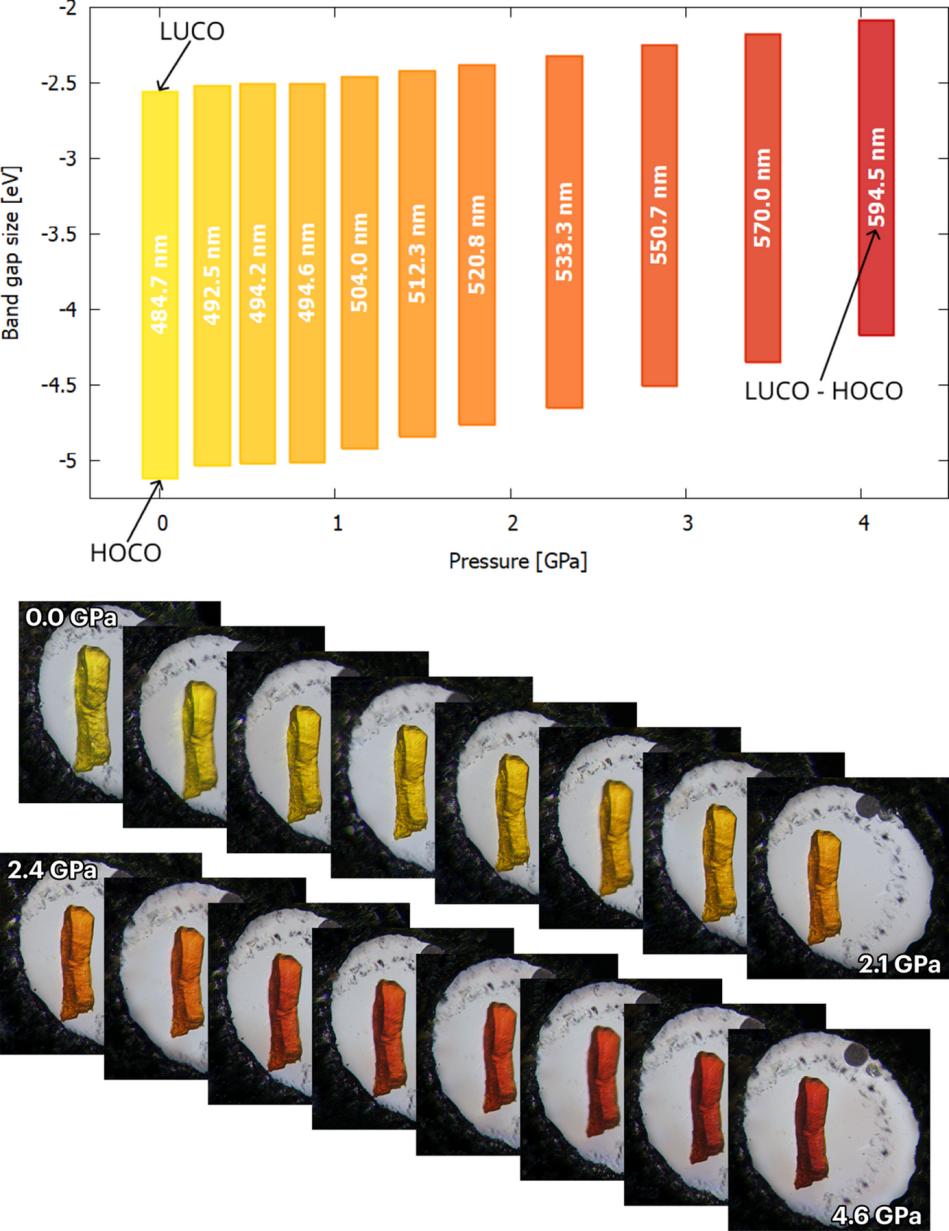
Changes in the geometry of π-stacking interactions in the structure for 2°AP-β with applied pressure result in shrinking of the band gap and consequently in a change of the absorbed wavelengths in the visible range. Maximum absorption wavelengths calculated from the HOCO–LUCO energy gap, and the resulting complementary colors observed for the crystal, are in excellent agreement.

As expected, compression of the π-stacks affects the energy gap between the highest occupied crystalline orbital (HOCO) and the lowest unoccupied crystalline orbital (LUCO), as confirmed by periodic DFT calculations. The energy gap, typical for a semiconductor under atmospheric conditions, decreases from 2.56 eV at 1 atm to 2.09 eV above 4.08 GPa (Fig. 8 ▸), which is mainly due to destabilization of the HOCO orbital by 0.60 eV. As a consequence, 2°AP-β displays significant piezochromism in the investigated pressure range (Fig. 8 ▸).

**Figure 9:**
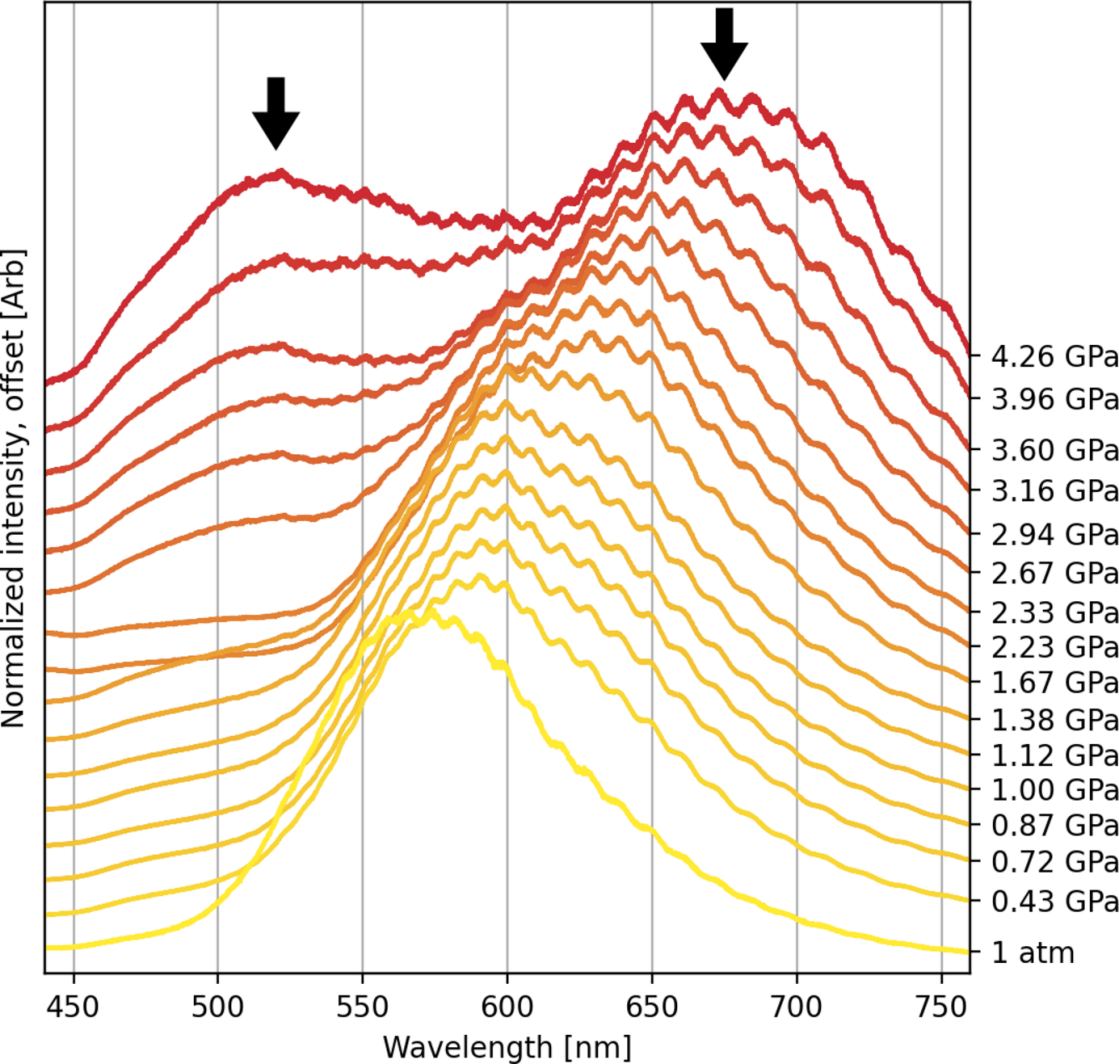
Shift of the luminescence maximum for 2°AP-β with increasing applied pressure. The formation of a new emission band at about 500 nm above 2.00 GPa suggests an onset of prominent lattice vibrations or structural changes yet to be investigated.

Closing the band gap with pressure necessarily affects the fluorescence of 2°AP-β, resulting in a pronounced shift of the emission maximum from 575 nm under atmospheric conditions to 685 nm above 4.08 GPa (Fig. 9 ▸). Somewhat unexpectedly, a second emission band appears at 2.31 GPa (roughly at the point where intermolecular interaction energies become less negative) and becomes more intense than the initial one as the pressure increases. It suggests either changes in the crystal structure of 2°AP-β or at least an onset of some new significant lattice oscillation mode. The reason for this change could not be established due to the lack of high-pressure diffraction data and it will be a subject of further investigation.

## 4. Conclusions

A new monoclinic polymorph of 1,3-diacetylpyrene (2°AP-β) has been characterized in a wide temperature and pressure range from structural and spectroscopic points of view. Crystallization from the melt yielded single crystals of satisfactory quality, suitable for analysis by quantum crystallography approaches. Most importantly, we proved that, by placing a crystal of 2°AP-β in a proper orientation in a wide-opening-angle DAC and applying a bright synchrotron X-ray source, it is possible to obtain high-quality and over 80%-complete high-pressure X-ray diffraction data for a weakly scattering low-symmetry organic sample. This, in turn, allowed us to perform unrestrained HAR refinements of the crystal structures, gaining reliable high-pressure structure geometries in excellent agreement with periodic DFT calculations. In particular, we performed a satisfactory and meaningful refinement of anharmonic thermal motions of carbonyl oxygen atoms at 0.85 GPa. Contrary to the previously reported 2°AP-α polymorph, the structure of 2°AP-β is based on well separated π-stacks of antiparallel molecules in the [100] direction. The presence of π-stacking translated to notable piezochromism and strong luminescence in the solid state, with an impressive 50 nm red-shift of the emission maximum from 1 atm to 4.08 GPa. This shift was accurately predicted by our periodic DFT calculations. The π-stacking also coincides with the direction of the largest compressibility in 2°AP-β. On the other hand, the apparently weaker C—H⋯O interactions indicate the direction of minimum compressibility and of a substantial NTE (−55.8 (57) MK^−1^), pointing to their importance. The trends observed in intermolecular interaction energies and an additional band appearing in the fluorescence spectra of 2°AP-β suggest an onset of a major structural reorganization above 2.00 GPa.

## 5. Related literature

The following references are cited in the supporting information: Groom *et al.* (2016 ▸); Macrae *et al.* (2008 ▸).

## Supplementary Material

Crystal structure: contains datablock(s) 2oAPb_100K, 2oAPb_293K_HAR, 2oAPb_293K_HAR_anh, 2oAPb_0p85GPa_HAR, 2oAPb_0p85GPa_HAR_anh, 2oAPb_1p1GPa_HAR, 2oAPb_1p7GPa_HAR. DOI: 10.1107/S2052252524003634/yc5047sup1.cif

Supporting figures and tables. DOI: 10.1107/S2052252524003634/yc5047sup2.pdf

CCDC references: 2336014, 2336015, 2336016, 2336017, 2336018, 2336019, 2336020

## Figures and Tables

**Table 1:** Crystallographic data and refinement statistics

Data collection					
Temperature (K)	100	293	298	298	298
Pressure	1 atm	1 atm	0.85 GPa	1.14 GPa	1.71 GPa
Space group	*P*2_1_/*c*	*P*2_1_/*c*	*P*2_1_/*c*	*P*2_1_/*c*	*P*2_1_/*c*
*a* (Å)	7.15110 (13)	7.2350 (3)	6.9685 (2)	6.90861 (18)	6.8196 (10)
*b* (Å)	10.90793 (19)	11.2573 (4)	10.7405 (4)	10.6550 (5)	10.5519 (14)
*c* (Å)	17.7744 (3)	17.7365 (5)	17.5252 (6)	17.4709 (7)	17.3617 (17)
β (°)	102.4944 (19)	103.701 (3)	101.697 (3)	101.448 (3)	100.735 (12)
*V* (Å^3^)	1353.63 (4)	1403.47 (9)	1284.44 (8)	1260.48 (8)	1227.5 (3)
λ (Å)	0.71073	1.54184	0.41618	0.41618	0.41618
Resolution (Å)	0.63	0.79	0.65	0.66	0.70
Potency (%)	100	100	93	93	94
Completeness (%)	99.8	99.1	81.9	86.2	90.1

Data refinement					
IAM/*SHELXL*					
*R*_1_ [*I* > 2σ(*I*)]	0.044	0.065	0.048	0.057	0.101
*wR*_2_ [*I* > 2σ(*I*)]	0.119	0.166	0.124	0.148	0.2735
Largest difference peak/hole (eÅ^−3^)	0.471/−0.194	0.297/−0.228	0.283/−0.179	0.330/−0.225	0.383/−0.433
DISCaMB/*NoSpherA2*					
*R*_1_ [*I* > 2σ(*I*)]	0.025	0.054	0.037	0.051	0.0996
*wR*_2_ [*I* > 2σ(*I*)]	0.039	0.116	0.076	0.125	0.2792
Largest difference peak/hole (eÅ^−3^)	0.241/−0.233	0.421/−0.294	0.2035/−0.2396	0.235/−0.327	0.427/−0.537
HAR/*NoSpherA2*					
*R*_1_ [*I* > 2σ(*I*)]	0.025	0.042	0.036	0.050	0.0990
*wR*_2_ [*I* > 2σ(*I*)]	0.039	0.094	0.074	0.124	0.2757
Largest difference peak/hole (eÅ^−3^)	0.240/−0.234	0.241/−0.194	0.203/−0.216	0.245/−0.324	0.475/−0.478
