# Supplementary material for: Evolution of structure and spectroscopic properties of a new 1,3-diacetylpyrene polymorph with temperature and pressure
Source: IUCrJ. 2024 May 10;11(Pt 4):519–27. doi: 10.1107/S2052252524003634 (PMC11220879; doi:10.1107/S2052252524003634)
Supplement: Supplementary file 2 [file m-11-00519-sup2.pdf]

IUCrJ

Volume 11 (2024)

Supporting information for article:

Evolution of structure and spectroscopic properties of a new 1,3-diacetylpyrene polymorph with temperature and pressure

A. Zwolenik, D. Tchoń and A. Makal

Evolution of structure and spectroscopic properties of a new 1,3-diacetylpyrene polymorph with temperature and pressure

Aleksandra Zwolenik, Daniel Tchoń and Anna Makal
contact: *am.makal@uw.edu.pl*

March 29, 2024

Contents

S1 Supporting materials **S2**

S1 Crystallization procedures S2

S2 Melting point determination S4

S3 Series of X-ray experiments S4

S3.1 X-ray diffraction experiments at atmospheric pressure S4

S3.2 High-pressure experiments S9

S4 Validation of HAR refinements S11

S5 Final crystallographic tables S14

S5.1 Deposition numbers S15

S6 Luminescence measurement details S15

S7 Periodic DFT details S15

S8 Geometry of 2°AP-β S17

S9 Thermal expansion and compressibility coefficients of 2°AP-β S19

S10 Intermolecular interactions energies of 2°AP-β S20

S2 Bibliography **S25**

Chapter S1

Supporting materials

S1 Crystallization procedures

Recrystallizations from several solvents and their mixtures have been attempted in order to obtain single crystals. In each case, a mixture of both polymorphs was obtained. Most crystallizations yielded light yellow, thin, hexagonal plates of 2°AP-α and orange adhesions of thin needles or very small blocks of 2°AP-β. The majority of crystals were growing on each other and were of small size, which made them impossible to separate and could only be distinguished by their luminescence.

The best way to obtain single crystals of 2°AP-β is to melt crystals of any 2°AP polymorph. For this experiment, LinkamScientific TMS94 heating table was used while placed under a microscope. Crystals were placed in a sealed glass vial and quickly (20°C/min) heated to 170°C. Then temperature was slowly (5°C/min) increased to 190°C. At this temperature, some crystals were starting to melt, but after waiting 5 minutes, there were still many that didn't. The temperature was slowly (5°C/min) increased to 195°C, and this temperature was held for another 10 minutes. At this temperature, small yellow particles were found at the top of the vial, which suggests the sublimation of this compound. The temperature was once again slowly (5°C/min) increased to 202°C and held for 10 minutes to make sure that all of the crystals had melted. After that, everything was quickly (20°C/min) cooled to room temperature. This procedure yielded large, orange, solidified chunks on the bottom of the vial and small, yellow particles on the walls in the upper part of the vial. After cutting the chunks from the bottom of the vial, smaller, irregular blocks were obtained and identified as single crystals of a new polymorphic form by X-ray diffraction.

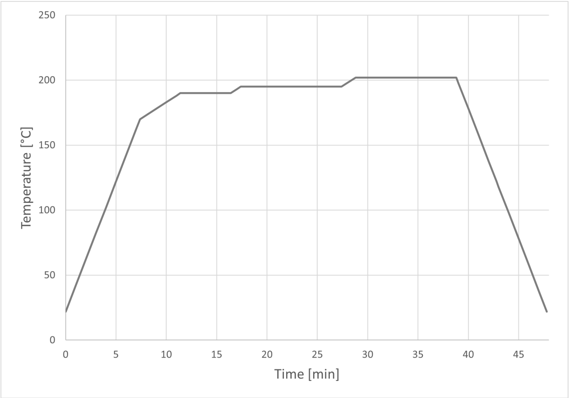

Figure S1.1: *An example heating / cooling protocol for obtaining single crystals of 2°AP-β.*

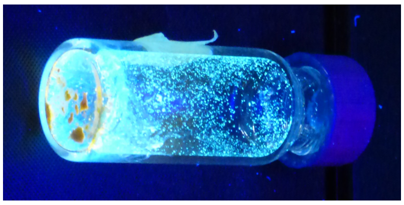

(a) *Vial after melting experiments.*

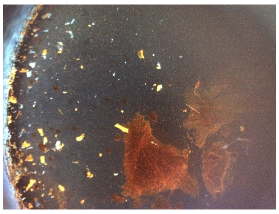

(b) *Crystals of 2°AP-β on the bottom of the vial.*

Figure S1.2: *The appearance of the vial and the crystals inside it after the melting experiment, observed under UV light (λ = 395 nm).*

Table S1.1: *List of solvents and their mixtures used for recrystallizations of 2°AP with qualitative solubility of this compound.*

Solvent	Solubility
acetone	good
ethyl acetate	moderate
acetic acid	moderate
toluene	good
furan	moderate
1,2,4-trichlorobenzene	good
diethyl ether	moderate
ethanol + acetone	poor + good
ethanol + toluene	poor + good
ethyl acetate + acetone	poor + good
ethyl acetate + toluene	poor + good
xylene + 1,2,4-trichlorobenzene	poor + good
n-pentane + acetone	poor + good
n-pentane + toluene	poor + good
n-pentane + 1,2,4-trichlorobenzene	poor + good
n-pentane + furan	poor + moderate

S2 Melting point determination

Due to the method of obtaining the 2°AP-β polymorph, determination of the melting point temperature was performed for a couple of single crystals at atmospheric pressure. The purity and quality of the crystal were confirmed by a short X-ray diffraction experiment (unit cell determination). Crystals were placed on a siliconized glass wafer on a LinkamScientific TMS94 hot stage and then slowly heated at a rate of 2°C/min in air under a microscope. Such a procedure was repeated twice on fresh single crystals. The melting point was determined at 174°C.

Melting points for polymorphs of 2°AP compound were also redetermined using the MP70 Melting Point System capillary apparatus (Mettler Toledo) with a heating rate of 5°C/min, yielding results within 1°C of the observations for single-crystals. The melting point temperatures observed for the formerly described α polymorphs of 1,3-diacetylpyrene isomers were in excellent agreement with the former observations by [Rajagopal et al., 2014] within 2°C.

S3 Series of X-ray experiments

S3.1 X-ray diffraction experiments at atmospheric pressure

For the variable temperature experiments, 5 crystals of 2°AP-β were used:

- crystal 1 - an orange single crystal of dimensions 0.130×0.185×0.216 mm, mounted on a glass capillary with a trace of epoxy resin.
- crystal 2 - a yellow single crystal of dimensions 0.112×0.206×0.279 mm, mounted on a MiTeGen loop.
- crystal 3 - a yellow single crystal of dimensions 0.093×0.135×0.175 mm, mounted on a nylon loop.
- crystal 4 - a yellow single crystal of dimensions 0.058×0.160×0.185 mm, mounted on a nylon loop with a trace of epoxy resin.
- crystal 5 - a yellow single crystal of dimensions 0.044×0.130×0.169 mm, mounted on a nylon loop with a trace of epoxy resin.

X-ray diffraction experiments at variable temperatures were performed using a Rigaku Oxford Diffraction Supernova diffractometer together with an Oxford Cryosystem 700 series temperature adapter filled with liquid nitrogen. The data were collected in CrysAlisPro [Rigaku-Oxford-Diffraction, 2019]. The following 10 different measurement series were performed using different radiations:

- using Cu Kα (λ = 1.54184 Å):
  - in 275 K (crystal 2)
  - every 10 K from 110 to 300 K (crystal 3)
  - every 10 K from 290 to 180 K (crystal 4)

  - every 10 K from 190 to 270 K (crystal 4)
  - every 10 K from 130 to 90 K (crystal 4)
  - in 293 K (crystal 5)

- using Mo Kα ($\lambda = 0.71073$ Å):
  - in 100K (crystal 1)
  - every 10 K from 300 to 390 K (crystal 4)
  - every 20 K from 370 to 290 K (crystal 4), (only unit-cell parameters scans)
  - every 10 K from 110 to 90 K (crystal 4)

After analyzing results from 110-300 K (Cu Kα) series, it was noted that unit cell constant **c** started decreasing with increasing temperature. To check the consistency of such anomalous behavior further experimental series involving cooling from 290 to 180 K and warming from 190 to 270 K (Cu Kα) were performed. After obtaining consistent results and for further analysis, the 300-390 K and 370-290 K (Mo Kα) series were performed. A setup with Mo Kα radiation source was chosen in order to allow high temperature measurements.

Another observation after analyzing results from 110-300 K (Cu Kα) series was a discrepancy between unit cell parameters obtained at 110 K and at 100K (Mo Kα) from formerly conducted experiment. In order to check the consistency of unit cell constants at low temperatures, the 110-90 K (Mo Kα) and 130-90 K (Cu Kα) series were performed. This time crystal was cooled down slowly (placed on a diffractometer at room temperature and cooled down in the same rate as cryosystem), as opposed to previous experiments, when crystal was placed directly under 100 K liquid nitrogen stream.

Three general types of experiments were performed:

- S - full structural experiment, fulfilling IUCr completeness criteria (around 99%), with over 2500 reflections,

- T - shorter experiments, with completeness in range of 50-80%, with approx. 1500-2000 reflections,

- P - short control pre-experiment, yielding less than 30 reflections.

Table S3.1: *Unit cell parameters of structures studied in multi-temperature diffraction measurements. The letter in the last column means respectively: S - structure on complete data; T - tentative structure on incomplete data; P - pre-experiment. Systematic extinctions consistent with $P2_1/c$ space group.*

T [K]	a [Å]	b [Å]	c [Å]	β [°]	V [Å^3]	
110-300 K, CuKα, Crystal 3						
110	7.14940(18)	10.9139(3)	17.7592(5)	102.584(3)	1352.42(6)	S
120	7.15390(18)	10.9223(3)	17.7632(5)	102.606(3)	1354.50(6)	S
130	7.15997(17)	10.9309(3)	17.7658(5)	102.654(3)	1356.66(6)	S
140	7.16439(17)	10.9383(3)	17.7682(5)	102.692(2)	1358.41(6)	S
150	7.17156(18)	10.9455(3)	17.7694(5)	102.742(3)	1360.48(6)	S
160	7.17601(18)	10.9558(3)	17.7734(5)	102.765(3)	1362.79(6)	S
170	7.18144(18)	10.9644(3)	17.7758(5)	102.791(3)	1364.94(6)	S
180	7.18713(18)	10.9759(3)	17.7771(5)	102.835(3)	1367.31(6)	S
190	7.19279(15)	10.9883(2)	17.7785(5)	102.878(2)	1369.8(5)	S
200	7.19749(16)	10.9995(3)	17.7789(5)	102.915(2)	1371.92(6)	S
210	7.20349(17)	11.0121(3)	17.7792(5)	102.950(2)	1374.47(6)	S
220	7.20794(19)	11.0291(3)	17.7743(5)	102.989(3)	1376.85(6)	S
230	7.21373(18)	11.0425(3)	17.7733(5)	103.013(2)	1379.42(6)	S
240	7.2195(2)	11.0619(3)	17.7779(5)	103.075(3)	1382.96(7)	T
250	7.2243(2)	11.085(3)	17.781(5)	103.130(2)	1386.7(7)	T
260	7.22709(19)	11.1105(3)	17.7724(5)	103.206(3)	1389.32(7)	T
270	7.2278(7)	11.1429(10)	17.776(2)	103.246(12)	1393.5(3)	T
280	7.2340(4)	11.1845(5)	17.7621(9)	103.403(2)	1397.98(12)	T
290	7.2317(4)	11.2284(6)	17.7446(9)	103.553(2)	1400.74(13)	T
300	7.2340(4)	11.2845(6)	17.7255(9)	103.782(2)	1405.31(13)	T
290-180 K, Cu Kα, Crystal 4						
290	7.2320(3)	11.2208(6)	17.7212(8)	103.576(4)	1397.87(12)	T
280	7.2318(2)	11.1766(4)	17.7468(6)	103.452(3)	1395.07(8)	T
270	7.2296(2)	11.1338(5)	17.7592(6)	103.333(3)	1390.96(9)	T
260	7.2250(2)	11.1042(5)	17.7607(6)	103.206(3)	1387.22(9)	T
250	7.2222(2)	11.0779(4)	17.7688(5)	103.146(3)	1384.37(8)	T
240	7.2169(2)	11.0607(4)	17.7699(6)	103.076(3)	1381.68(8)	T
230	7.2136(2)	11.0431(4)	17.7697(5)	103.057(3)	1378.95(8)	T
220	7.2080(2)	11.0272(4)	17.7693(5)	103.006(3)	1376.14(8)	T
210	7.2039(2)	11.0096(4)	17.7763(5)	102.981(3)	1373.85(8)	T
200	7.1977(2)	10.9944(3)	17.7718(5)	102.904(2)	1370.84(7)	T
190	7.1920(2)	10.9858(3)	17.7753(5)	102.894(2)	1369.01(7)	T
180	7.1866(2)	10.9731(3)	17.776(5)	102.846(2)	1366.72(7)	T

Table S3.2: *Unit cell parameters of structures studied in multi-temperature diffraction measurements. The letter in the last column means respectively: S - structure on complete data; T - tentative structure on incomplete data; P - pre-experiment. Systematic extinctions consistent with $P2_1/c$ space group.*

T [K]	a [Å]	b [Å]	c [Å]	β [°]	V [Å^3]	
190-270 K, Cu Kα, Crystal 4						
190	7.1920(2)	10.9868(3)	17.7712(5)	102.868(2)	1368.96(7)	T
200	7.1977(2)	10.9992(3)	17.7724(5)	102.901(2)	1371.51(7)	T
210	7.2026(2)	11.0132(3)	17.7762(5)	102.958(2)	1374.17(7)	T
220	7.2083(2)	11.0283(3)	17.7737(5)	103.01(2)	1376.66(7)	T
230	7.2128(2)	11.0418(4)	17.7775(5)	103.048(3)	1379.29(8)	T
240	7.2184(2)	11.0564(3)	17.7762(5)	103.091(2)	1381.84(7)	T
250	7.2225(2)	11.0785(4)	17.7738(5)	103.157(3)	1384.83(8)	T
260	7.2253(2)	11.1022(4)	17.7722(5)	103.23(3)	1387.79(8)	T
270	7.2278(2)	11.1381(4)	17.7645(6)	103.336(3)	1391.55(8)	T
300-390 K, Mo Kα, Crystal 4						
300	7.2332(8)	11.2713(11)	17.734(3)	103.754(13)	1404.4(3)	T
310	7.2376(9)	11.3305(12)	17.708(3)	103.926(14)	1409.4(3)	T
320	7.2393(10)	11.3720(13)	17.702(3)	104.107(15)	1413.3(4)	T
330	7.2412(10)	11.4111(13)	17.696(3)	104.219(14)	1417.5(4)	T
340	7.2481(11)	11.4455(13)	17.698(3)	104.303(16)	1422.6(4)	T
350	7.2559(13)	11.4808(15)	17.713(3)	104.455(18)	1428.9(4)	T
360	7.2793(12)	11.5072(13)	17.723(3)	104.474(17)	1437.4(4)	T
370	7.2910(14)	11.537(16)	17.748(4)	104.65(2)	1444.4(5)	T
380	7.2946(18)	11.550(2)	17.755(5)	104.65(3)	1447.3(6)	T
390	7.273(3)	11.567(3)	17.710(6)	104.44(4)	1443.0(8)	T
370-290 K, Mo Kα, Crystal 4						
370	7.278(16)	11.543(16)	17.71(5)	104.7(2)	1440(6)	P
350	7.268(15)	11.448(10)	17.684(19)	104.65(12)	1424(4)	P
330	7.252(16)	11.441(16)	17.63(3)	104.16(16)	1419(5)	P
310	7.251(14)	11.37(2)	17.69(5)	104.05(18)	1415(6)	P
290	7.261(15)	11.249(12)	17.72(3)	103.71(16)	1406(4)	P

Table S3.3: *Unit cell parameters of structures studied in multi-temperature diffraction measurements. The letter in the last column means respectively: S - structure on complete data; T - tentative structure on incomplete data; P - pre-experiment. Systematic extinctions consistent with* $P2_1/c$ *space group.*

T [K]	a [Å]	b [Å]	c [Å]	β [°]	V [Å^3]	
130-90 K, Cu Kα, Crystal 4						
130	7.16135(12)	10.93188(16)	17.7683(3)	102.6408(16)	1357.31(4)	S
120	7.15924(11)	10.92722(16)	17.7681(3)	102.6297(15)	1356.38(4)	S
110	7.15870(11)	10.92602(16)	17.7642(3)	102.6181(15)	1355.89(4)	S
100	7.15830(11)	10.92705(15)	17.765(3)	102.6133(15)	1356.02(4)	T
90	7.15838(11)	10.92582(15)	17.7652(3)	102.6203(14)	1355.87(4)	S
110-90 K, Mo Kα, Crystal 4						
110	7.1506(5)	10.9137(5)	17.7692(19)	102.518(8)	1353.73(18)	T
100	7.1454(4)	10.9031(5)	17.7684(18)	102.493(7)	1351.5(17)	T
90	7.1403(4)	10.8959(5)	17.7691(7)	102.445(7)	1349.95(17)	T
100 K, Mo Kα, Crystal 1						
100	7.15110(13)	10.90793(19)	17.7744(3)	102.4944(19)	1353.63(4)	S
275 K, Cu Kα, Crystal 2						
275	7.22800(1)	11.1585(2)	17.7576(4)	103.374(2)	1393.37(5)	S
293 K, Cu Kα, Crystal 5						
293	7.2350(3)	11.2573(4)	17.7365(5)	103.701(3)	1403.47(9)	S

S3.2 High-pressure experiments

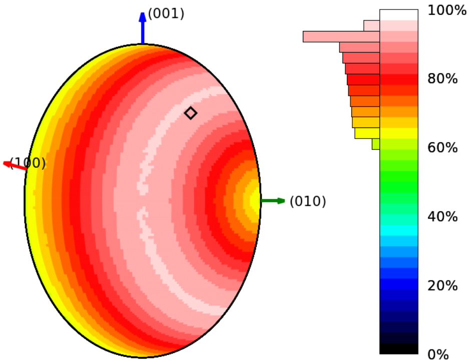

Figure S3.1: *Map of all possible crystal orientations with histogram of completenesses that can be theoretically achieved for our monoclinic sample with DAC opening angle of 52° and radiation wavelength $\lambda = 0.41618$ Å [Tchoń and Makal, 2021]. Theoretically predicted completeness of a sample placed in DACOne20 in the actual experimental orientation is 93% (marked by black square).*

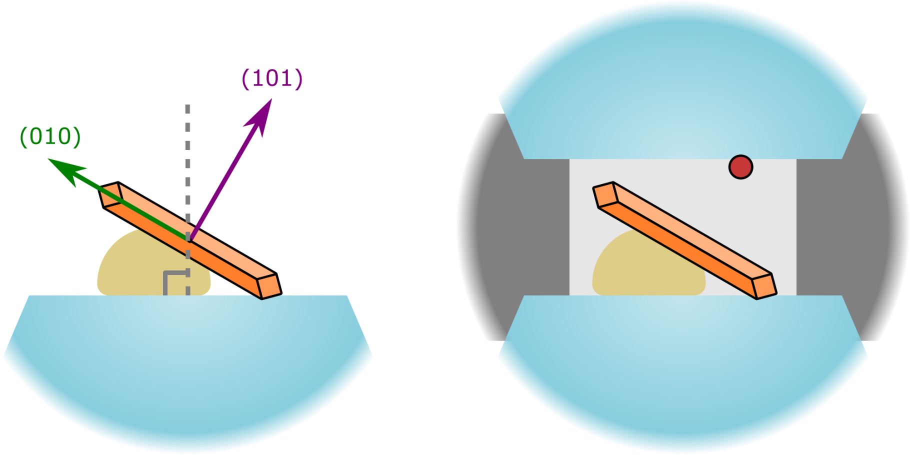

(a) *Before closing.* ***(b)*** *After closing.*

Figure S3.2: *The strategy employed to place the crystal in a favorable orientation. According to the potency map (Figure S3.1) for the monoclinic sample at the given experimental conditions, maximal data coverage can be obtained when (100), (001) and (010) directions are not aligned with the DAC axis. (a) A small droplet of the epoxy resin (light yellow) is made on one culet of the DAC (blue) and left to dry. The crystal (orange), after preliminary face-indexing, is propped up to avoid alignment of the crystallographic directions with the DAC main axis, i.e. to avoid (100), (001) and (010) crystal faces being parallel to the diamond face. A trace amount of fresh glue might be used to prevent the crystal from sliding. (b) The DAC is then furnished with the gasket (gray), ruby chip (red), filled with pressure-transmitting medium (light-gray), and closed in preparation for an XRD experiment.*

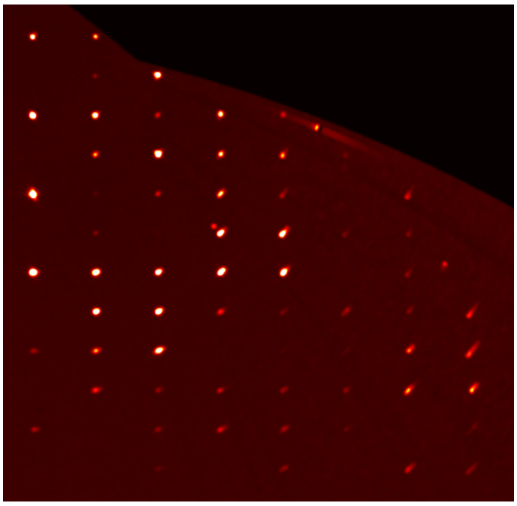

(a) Layer 1kl for 0.85 GPa.

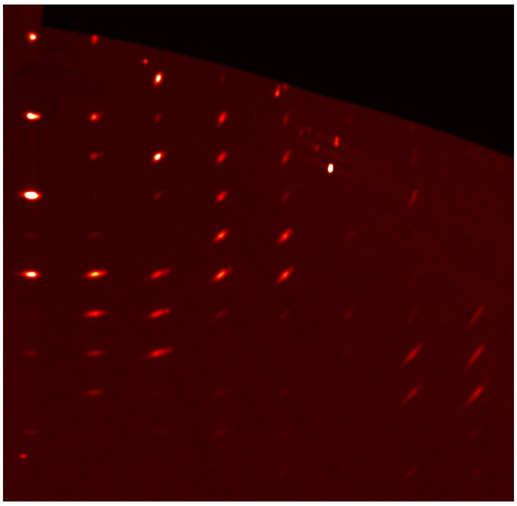

(b) Layer 1kl for 1.7 GPa.

Figure S3.3: *Layers 1kl for high-pressure experiments in 0.85 and 1.7 GPa. Decrease of intensity and elongation of reflections indicate sample detoriation.*

Table S3.4: *Unit cell parameters of structures under applied pressure with included reference structure from 293 K. The letter in the last column means respectively: S - solved structure; P - pre-experiment.*

p [GPa]	a [Å]	b [Å]	c [Å]	β [°]	V [Å^3]	
Rigaku Oxford Diffraction Supernova						
1 atm	7.2350(3)	11.2573(4)	17.7365(5)	103.701(3)	1403.47(9)	S
0.30(10)	7.090(4)	10.880(6)	17.70(3)	102.4(1)	1333(3)	P
0.44(10)	7.037(9)	10.775(9)	17.58(10)	102.6(3)	1301(8)	P
0.71(10)	7.045(10)	10.850(15)	17.44(6)	101.1(2)	1309(5)	P
1.34(10)	6.915(10)	10.725(12)	17.35(7)	101.2(2)	1263(5)	P
1.42(10)	6.848(7)	10.672(14)	17.34(8)	101.1(2)	1243(6)	P
1.59(10)	6.819(5)	10.674(9)	17.30(5)	100.94	1237(4)	P
SOLEIL						
0.85(10)	6.9685(2)	10.7405(4)	17.5252(6)	101.697(3)	1284.44(8)	S
1.14(10)	6.9086(2)	10.6550(5)	17.4709(7)	101.448(3)	1260.48(8)	S
1.71(10)	6.8196(1)	10.5519(14)	17.3617(17)	100.735(12)	1227.5(3)	S

S4 Validation of HAR refinements

harmonic:

anharmonic:

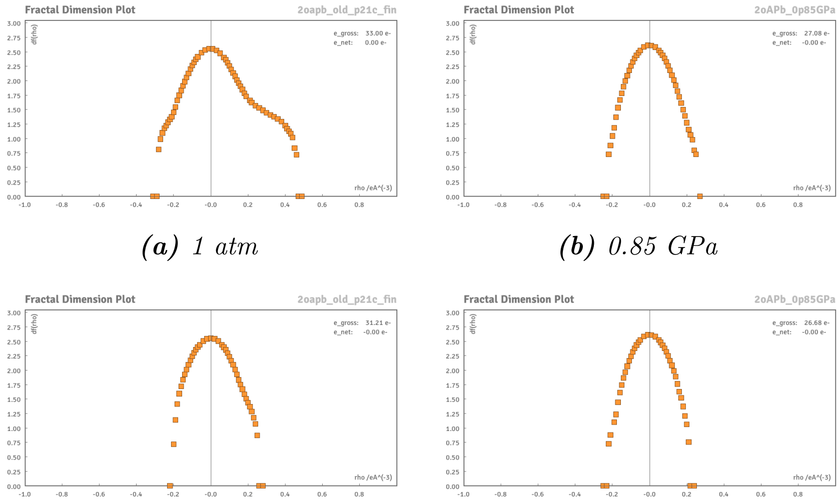

(a) *1 atm* ***(b)*** *0.85 GPa*

(c) *1 atm* ***(d)*** *0.85 GPa*

Figure S4.1: *Fractal dimension plots [Meindl and Henn, 2008] for structures with and without anharmonic atomic displacement parameters refined. Ideal parabolic shape suggests a random distribution of differential density, i.e. a well-fitting structure model.*

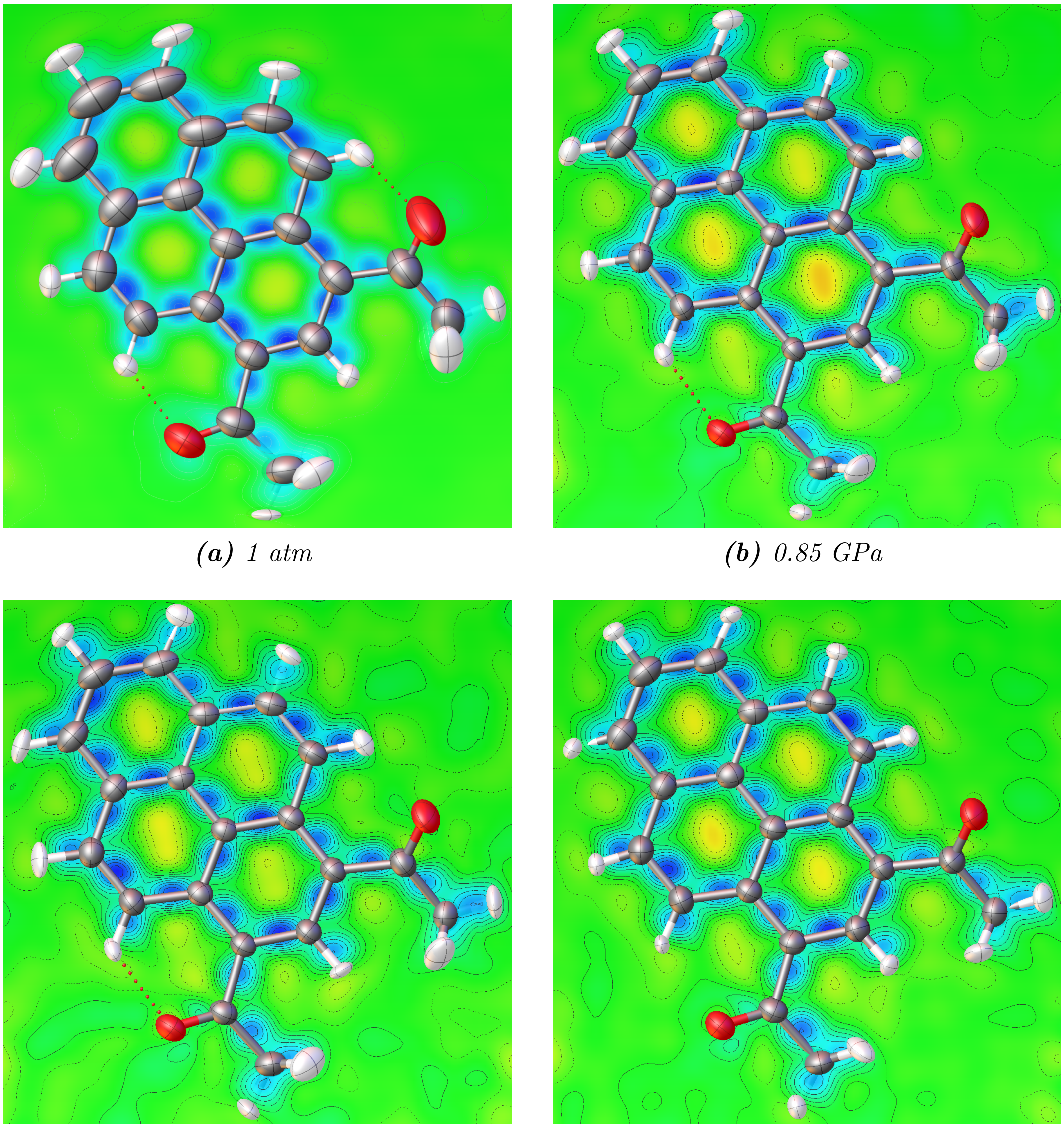

(a) *1 atm*

(b) *0.85 GPa*

(c) *1.14 GPa*

(d) *1.71 GPa*

Figure S4.2: *Dynamical deformation density maps generated in Olex2 [Dolomanov et al., 2009] after HAR refinement with anharmonic atomic displacement parameters where applicable.*

Table S4.1: *A listing of refined Gramm-Charlier parameters describing correction for anharmonicity in thermal motions of O1 and O2 atoms at 1 atm and 0.9 GPa. Statistically significant coefficients are represented in bold.*

	O1		O2	
3-rd order	1 atm	0.85 GPa	1 atm	0.85 GPa
GC_C_111	**-0.00007(2)**	**-0.000012(4)**	**-0.000187(10)**	**-0.0000157(17)**
GC_C_112	-0.000005(6)	0.0000001(12)	**0.000045(3)**	0.0000013(6)
GC_C_113	0.000002(4)	0.0000007(8)	**-0.000026(2)**	0.0000001(4)
GC_C_122	0.000001(2)	-0.0000000(7)	**-0.0000088(11)**	-0.0000001(3)
GC_C_123	0.0000002(13)	0.0000003(3)	**0.0000064(6)**	0.00000030(14)
GC_C_133	-0.0000006(12)	0.0000001(3)	**-0.0000048(6)**	0.00000011(14)
GC_C_222	-0.0000002(16)	-0.0000009(9)	0.0000015(7)	-0.0000001(4)
GC_C_223	0.0000001(7)	0.0000000(2)	-0.0000006(3)	-0.00000020(11)
GC_C_233	0.0000001(5)	0.00000006(16)	**0.0000011(2)**	0.00000006(7)
GC_C_333	-0.0000000(6)	-0.00000002(19)	-0.0000008(3)	0.00000001(9)
4-th order	1 atm	0.85 GPa	1 atm	0.85 GPa
GC_D_1111	0.000003(3)	0.0000006(5)	0.0000015(14)	0.0000006(3)
GC_D_1112	0.0000004(8)	-0.00000009(16)	-0.0000011(4)	-0.00000012(7)
GC_D_1113	-0.0000001(6)	0.00000004(10)	0.0000001(3)	0.00000012(5)
GC_D_1122	0.0000001(3)	-0.00000000(8)	0.00000036(14)	-0.00000000(4)
GC_D_1123	-0.00000002(16)	-0.00000001(3)	-0.00000017(8)	-0.000000019(15)
GC_D_1133	0.00000004(15)	0.00000001(3)	0.00000020(8)	0.000000033(15)
GC_D_1222	-0.00000007(16)	0.00000001(6)	-0.00000008(7)	-0.00000001(3)
GC_D_1223	0.00000003(7)	0.000000009(19)	0.00000003(3)	0.000000001(9)
GC_D_1233	-0.00000001(5)	-0.000000007(11)	-0.00000008(3)	-0.000000003(5)
GC_D_1333	0.00000001(6)	-0.000000007(14)	**0.00000014(3)**	0.000000008(7)
GC_D_2222	-0.00000004(14)	-0.00000011(8)	0.00000010(6)	**-0.00000014(4)**
GC_D_2223	0.00000001(5)	-0.000000002(18)	0.00000002(2)	0.000000003(8)
GC_D_2233	0.00000002(3)	0.000000007(10)	0.000000014(15)	0.000000006(5)
GC_D_2333	-0.00000002(3)	-0.000000003(7)	-0.000000035(13)	-0.000000001(3)
GC_D_3333	0.00000002(4)	-0.000000008(10)	**0.000000061(18)**	-0.000000012(5)

S5 Final crystallographic tables

Table S5.1: *Crystallographic data and performed refinements.*

Temperature [K]	100	293	298	298	298
Pressure	1 atm	1 atm	0.85 GPa	1.14 GPa	1.71 GPa
Empirical formula	$C_{20}H_{14}O_2$	$C_{20}H_{14}O_2$	$C_{20}H_{14}O_2$	$C_{20}H_{14}O_2$	$C_{20}H_{14}O_2$
Formula weight [$\frac{g}{mol}$]	286.31	286.31	286.31	286.31	286.31
Opening angle [°]	not applicable	not applicable	52	52	52
Crystal system	monoclinic	monoclinic	monoclinic	monoclinic	monoclinic
Space group	$P2_1/c$	$P2_1/c$	$P2_1/c$	$P2_1/c$	$P2_1/c$
a [Å]	7.15110(13)	7.2350(3)	6.9685(2)	6.90861(18)	6.8196(10)
b [Å]	10.90793(19)	11.2573(4)	10.7405(4)	10.6550(5)	10.5519(14)
c [Å]	17.7744(3)	17.7365(5)	17.5252(6)	17.4709(7)	17.3617(17)
α [°]	90	90	90	90	90
β [°]	102.4944(19)	103.701(3)	101.697(3)	101.448(3)	100.735(12)
γ [°]	90	90	90	90	90
Volume [Å^3]	1353.63(4)	1403.47(9)	1284.44(8)	1260.48(8)	1227.5(3)
Z, Z'	4, 1	4, 1	4, 1	4, 1	4, 1
ρ_{calc} [$\frac{g}{cm^3}$]	1.405	1.355	1.481	1.509	1.549
μ [mm^{-1}]	0.090	0.688	0.041	0.042	0.043
F(000)	600	600	600	600	600
Crystal size [mm^3]	0.17×0.14×0.04	0.17×0.13×0.04	0.25×0.15×0.10	0.25×0.15×0.11	0.20×0.15×0.11
Radiation source	Mo Kα	Cu Kα	synchrotron	synchrotron	synchrotron
Radiation wavelength [Å]	0.71073	1.54184	0.41618	0.41618	0.41618
2θ range	6.652 to 69.134	9.384 to 155.216	3.494 to 37.362	3.522 to 36.754	3.596 to 34.58
Reflections collected	48392	9165	33624	25886	20814
R_{int}	0.0488	0.0320	0.0828	0.0941	0.1252
Resolution [Å]	0.6263	0.7893	0.6497	0.6600	0.7001
Completeness [%]	99.8	99.1	81.9	86.2	90.1
Potency [%]	100	100	93	93	94
			IAM / SHELXL		
Data; restraints; parameters	5486; 0; 255	2913; 0; 201	3765; 0; 325	3841; 0; 325	3277; 0; 255
R_1 ($I > 2\sigma(I)$)	0.0440	0.0652	0.0485	0.0573	0.1010
wR_2 ($I > 2\sigma(I)$)	0.1198	0.1663	0.1240	0.1480	0.2735
R_1 (all data)	0.0631	0.0808	0.0735	0.0876	0.1551
wR_2 (all data)	0.1293	0.1772	0.1416	0.1741	0.3212
Largest diff. peak [eÅ^{-3}]	0.471	0.297	0.283	0.330	0.383
Largest diff. hole [eÅ^{-3}]	-0.194	-0.228	-0.179	-0.225	-0.433
			DISCaMB / NoSpherA2		
Data; restraints; parameters	5486; 0; 325	2912; 0; 325	3765; 0; 325	3841; 0; 325	3277; 0; 255
R_1 ($I > 2\sigma(I)$)	0.0254	0.0542	0.0370	0.0505	0.0996
wR_2 ($I > 2\sigma(I)$)	0.0390	0.1158	0.0758	0.1258	0.2792
R_1 (all data)	0.0449	0.0696	0.0628	0.0816	0.1538
wR_2 (all data)	0.0420	0.1239	0.0872	0.1521	0.3383
Largest diff. peak [eÅ^{-3}]	0.2415	0.4209	0.2035	0.2352	0.4279
Largest diff. hole [eÅ^{-3}]	-0.2334	-0.2939	-0.2396	-0.3278	-0.5378
			HAR / NoSpherA2		
Data; restraints; parameters	5486; 0; 325	2912; 0; 375	3765; 0; 325	3841; 0; 325	3269; 186; 326
R_1 ($I > 2\sigma(I)$)	0.0254	0.0420	0.0364	0.0506	0.0990
wR_2 ($I > 2\sigma(I)$)	0.0391	0.0947	0.0740	0.1249	0.2757
R_1 (all data)	0.0449	0.0572	0.0622	0.0816	0.1533
wR_2 (all data)	0.0421	0.1037	0.0851	0.1508	0.3359
Largest diff. peak [eÅ^{-3}]	0.2407	0.2413	0.2036	0.2454	0.4758
Largest diff. hole [eÅ^{-3}]	-0.2340	-0.1948	-0.2165	-0.3246	-0.4787

S5.1 Deposition numbers

Structures determined for the purpose of this paper were deposited as individual entries within Cambridge Structural Database [Groom et al., 2016]. Exact deposition number for each structure have been presented in Table S5.2.

Table S5.2: *CCDC deposition numbers of crystal structures determined for the purpose of these studies, sorted by crystal phase and exerted pressure.*

Temperature	Pressure	Atomic displacement model for oxygen atoms	CCDC deposition number
100 K	1 atm	harmonic	2336017
293 K	1 atm	harmonic	2336020
293 K	1 atm	anharmonic	2336014
298 K	0.85 GPa	harmonic	2336019
298 K	0.85 GPa	anharmonic	2336015
298 K	1.14 GPa	harmonic	2336018
298 K	1.71 GPa	harmonic	2336016

S6 Luminescence measurement details

The steady-state UV-vis fluorescence spectra were recorded with a Labram HR800 (Horiba JobinYvon) spectrometer coupled with an Olympus BX61 confocal microscope and equipped with a Peltier-cooled CCD detector (1024 $\times$ 256 pixel). A diode pumped, frequency doubled Nd:YAG laser 405 nm (output power 100 mW) was used as the excitation source. Calibration of the instrument was performed using a 520 cm^{-1} Raman signal of a silicon wafer. The spectra were collected in the 1atm - 4.3 GPa pressure range for the same single crystal specimen in a DAC which was used in *in-house* x-ray diffraction experiments.

S7 Periodic DFT details

Periodic calculations were made in Crystal17 [Dovesi et al., 2018], using the DFT method with B3LYP functional with the Grimme dispersion correction (D3) and the 6-311G basis set. In all cases, strict truncation criteria for the Coulomb and Exchange sums (10^{-7}, 10^{-7}, 10^{-7}, 10^{-7}, 10^{-29}) were applied to ensure proper convergence of the calculations.

The experimental geometry of the 275 K structure was optimized without temperature restraints. Starting from the same geometry, EOS calculations were also performed, producing 10 theoretical structures in pressure range from 0.06 to 4.08 GPa. For each structure single-point calculations were performed in order to obtain band gaps spans and lattice energies. Interaction energy calculations, taking into account the BSSE correction, were performed for molecules forming π-stacking interactions.

Table S7.1: *Unit cell parameters of structures predicted by periodic DFT calculations under applied pressure.*

p [GPa]	a [Å]	b [Å]	c [Å]	β [°]	V [Å^3]
1 atm, 275 K	6.7972	10.5601	17.8128	100.716	1256.30
0.06	7.0374	11.1413	18.0696	102.124	1385.17
0.30	7.0238	10.7842	18.3604	102.766	1356.35
0.56	6.9658	10.7030	18.2063	101.969	1327.86
0.84	6.8983	10.6414	18.0086	100.523	1299.73
1.14	6.8304	10.6181	17.8631	100.928	1272.04
1.47	6.7595	10.5582	17.7530	100.753	1244.76
1.81	6.7002	10.4406	17.7137	100.486	1218.45
2.31	6.6095	10.4000	17.5003	100.092	1184.34
2.85	6.5142	10.3308	17.3578	99.896	1150.75
3.44	6.4207	10.2559	17.2185	99.569	1118.06
4.09	6.3098	10.2004	17.0879	99.060	1086.09

Table S7.2: *Values of RMSD and maximal difference, calculated in Mercury [Macrae et al., 2008] in "molecule overlay" mode, of molecules, which were obtained experimentally and theoretically predicted.*

Experimental structure	Theoretical structure	RMSD [Å]	Maximal difference [Å]
100 K	optimized 275 K	0.0337	0.0915
0.85 GPa	0.84 GPa	0.0635	0.1742
1.14 GPa	1.14 GPa	0.0638	0.1728
1.71 GPa	1.81 GPa	0.0583	0.1534

Table S7.3: *Energies of frontier crystalline orbitals, resulting band gap spans and related absorption wavelengths, based on periodic DFT calculations.*

Pressure [GPa]	HOCO energy [eV]	LUCO energy [eV]	Band gap size [eV]	[nm]
1 atm	-5.228	-2.670	-2.558	484.66
0.30	-5.201	-2.684	-2.517	492.51
0.56	-5.168	-2.660	-2.509	494.22
0.84	-5.121	-2.615	-2.507	494.61
1.14	-5.091	-2.632	-2.460	504.00
1.36	-5.063	-2.609	-2.454	505.28
1.47	-5.046	-2.626	-2.420	512.29
1.81	-4.994	-2.613	-2.381	520.77
2.31	-4.924	-2.560	-2.324	533.32
2.85	-4.840	-2.588	-2.252	550.66
3.44	-4.744	-2.569	-2.175	570.00
4.08	-4.624	-2.539	-2.085	594.49

S8 Geometry of 2°AP-β

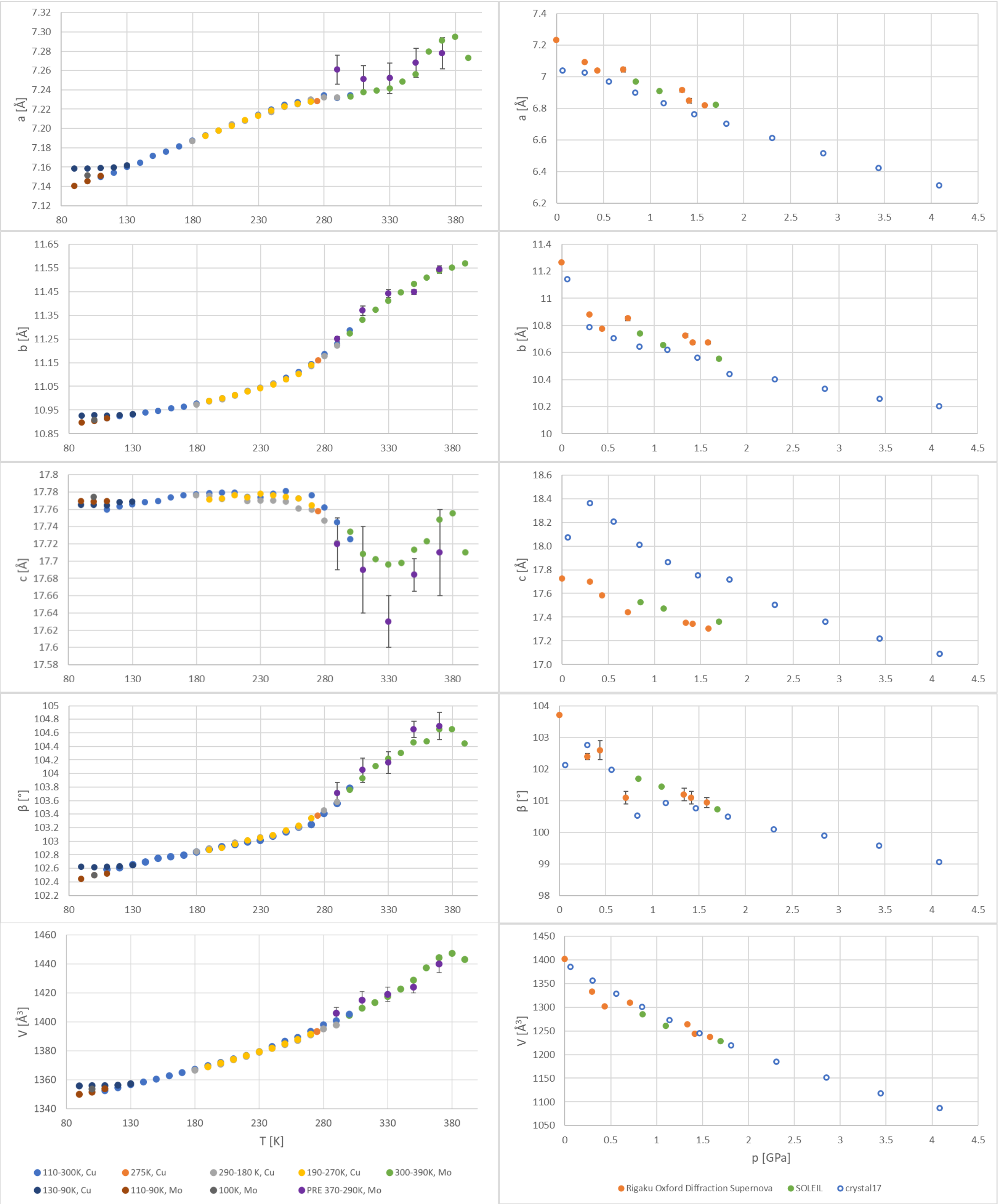

Figure S8.1: *Evolution of the unit cell parameters with external stimuli. Purple points, signed as PRE, were determined from the short pre-experiments (P), based on less than 30 reflections at each temperature point; this resulted in substantial uncertainties.*

Table S8.1: *Geometrical parameters describing dimers in the structure of 2°AP-β.*

Pressure [GPa]	Dimer [Å] plane-plane	Dimer [Å] plane-centroid	Dimer [Å] shift of centroids
Theoretical structures			
0.06	3.4115	3.6774	1.3731
0.30	3.4002	3.6504	1.3281
0.56	3.3744	3.6378	1.3592
0.84	3.3431	3.6303	1.4151
1.14	3.3114	3.5863	1.3772
1.81	3.2499	3.5400	1.4035
2.30	3.2105	3.5098	1.4183
2.85	3.1694	3.4707	1.4144
3.44	3.1274	3.4325	1.4147
4.08	3.0842	3.3956	1.4203
Experimental structures			
1 atm, 275 K	3.4478(4)	3.7080(4)	1.3646(6)
0.85	3.3812(4)	3.6460(4)	1.3640(6)
1.14	3.3511(7)	3.6172(6)	1.3617(10)
1.71	3.3060(18)	3.5786(17)	1.370(2)
**Pressure [GPa]**	**Between dimers [Å] plane-plane**	**plane-centroid**	**shift of centroids**
Theoretical structures			
0.06	3.5756	3.9779	1.7433
0.30	3.5629	3.9801	1.7739
0.56	3.5257	3.9098	1.6899
0.84	3.4809	3.8176	1.5675
1.14	3.4479	3.7897	1.5729
1.81	3.3656	3.7165	1.5764
2.30	3.3135	3.6639	1.5638
2.85	3.2589	3.6123	1.5584
3.44	3.2048	3.5579	1.5454
4.08	3.1441	3.4911	1.5175
Experimental structures			
1 atm, 275 K	3.6270(5)	4.0328(4)	1.7631(6)
0.85	3.5073(4)	3.8797(5)	1.6586(7)
1.14	3.4754(6)	3.8386(7)	1.6299(10)
1.71	3.4242(17)	3.7659(18)	1.567(3)

Table S8.2: *Selected dihedral angles for molecule in 2°AP-α [Rajagopal et al., 2014] and currently analyzed 2°AP-β polymorphs.*

	2°AP-α	2°AP-β				
	293 K	100 K	293 K	0.85 GPa	1.14 GPa	1.71 GPa
C2-C1-C17-O1	-32.8(1)	-17.41(6)	-17.0(3)	-18.2(1)	-18.4(2)	-18.3(5)
C12-C13-C19-O2	32.8(1)	23.79(6)	20.9(3)	25.8(1)	25.5(2)	27.2(5)
pyrene bend	0.87(5)	3.755(14)	3.33(6)	4.00(3)	4.07(4)	4.15(11)

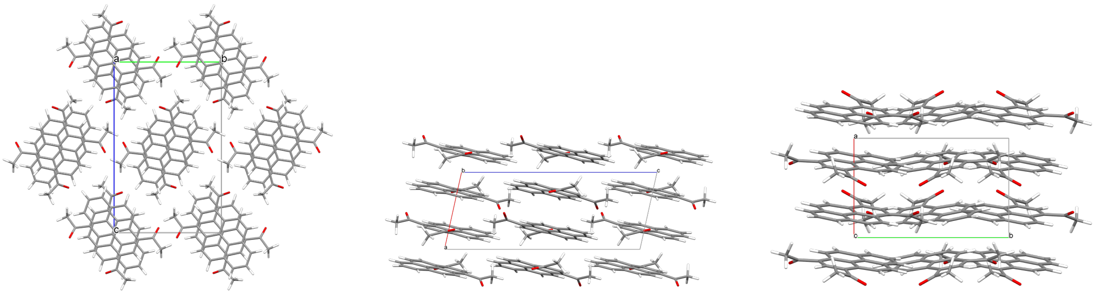

(a) *view along $\vec{a}$* ***(b)*** *view along $\vec{b}$* ***(c)*** *view along $\vec{c}$*

Figure S8.2: *Packing of molecules in structure of 2°AP-β.*

S9 Thermal expansion and compressibility coefficients of 2°AP-β

Table S9.1: *Thermal expansion α (MK^{-1}), based on experimental unit cell constants, calculated using PASCal Server [Cliffe and Goodwin, 2012].*

Main axis	Temperature expansion			
	Value [MK^{-1}]	Direction *a*	*b*	*c*
X1	-55.8(57)	-0.7688	0.0000	-0.6394
X2	84.6(31)	-0.9929	0.0000	0.1190
X3	192(13)	0.0000	1.0000	-0.0000

Table S9.2: *Compressibility K (TPa^{-1}) coefficients of 2°AP-β, based on experimental unit cell constants (left) and theoretical calculations (right), calculated using PASCal Server [Cliffe and Goodwin, 2012].*

Main axis	Experimental compressibility				Theoretical compressibility			
	Value [TPa^{-1}]	Direction *a*	*b*	*c*	Value [TPa^{-1}]	Direction *a*	*b*	*c*
X1	24(4)	-0.9868	0.0000	0.1621	24.9(14)	0.9638	0.0000	-0.2665
X2	18(9)	0.0000	-1.0000	0.0000	13(1)	0.0000	-1.0000	0.0000
X3	12(7)	-0.8203	0.0000	-0.5719	14.7(15)	-0.9052	0.0000	-0.4249

S10 Intermolecular interactions energies of 2°AP-β

Table S10.1: *Intermolecular interactions energies between molecules within π-stacks.*

Pressure [GPa]	Lattice energy [kJ/mol]	Interaction energy [kJ/mol]			
		Dimer		Between dimers	
		Crystal17	CrystalExplorer	Crystal17	CrystalExplorer
Theoretical structures					
0.06	-169.83	-75.53	-75.6	-66.41	-65.7
0.30	-172.32	-75.14	-75.5	-66.50	-65.7
0.56	-174.53	-75.77	-76.2	-67.55	-66.4
0.84	-175.65	-76.54	-77.0	-68.32	-66.9
1.14	-175.63	-77.70	-79.0	-68.88	-68.7
1.81	-171.83	-78.90	-79.7	-68.89	-67.1
2.30	-165.90	-79.10	-80.1	-68.09	-66.0
2.85	-156.74	-78.44	-79.7	-66.70	-63.9
3.44	-143.82	-76.81	-78.1	-64.19	-60.4
4.08	-126.06	-74.17	-75.3	-60.11	-54.9
Experimental structures					
0.85	-179.63	-78.33	-78.3	-67.53	-65.9
1.14	-180.05	-79.98	-80.1	-67.77	-65.9
1.71	-183.59	-82.58	-84.8	-66.98	-65.0

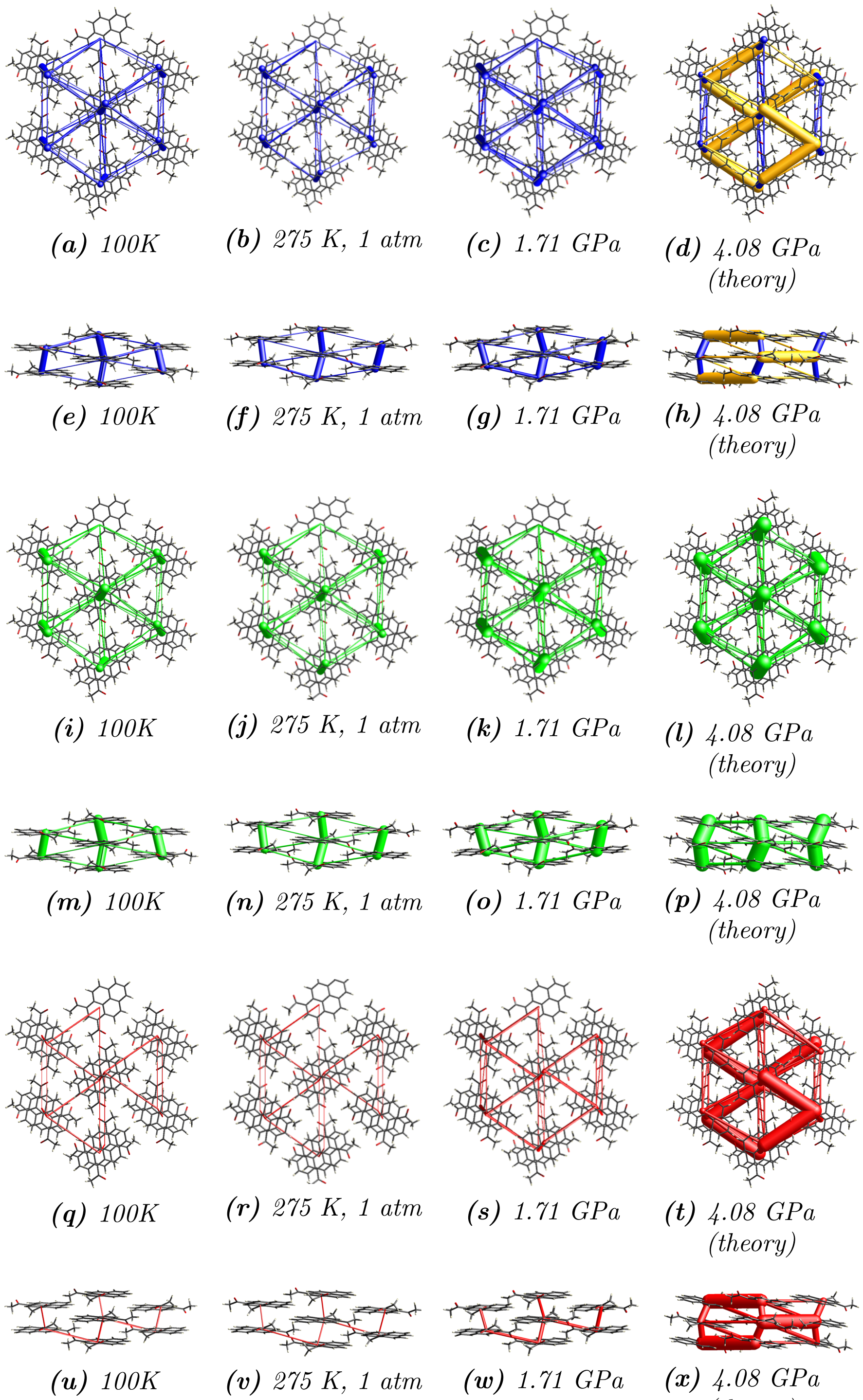

Total energy, view along $\vec{a}$:

(a) *100K* ***(b)*** *275 K, 1 atm* ***(c)*** *1.71 GPa* ***(d)*** *4.08 GPa (theory)*

Total energy, view along $\vec{b}$:

(e) *100K* ***(f)*** *275 K, 1 atm* ***(g)*** *1.71 GPa* ***(h)*** *4.08 GPa (theory)*

Dispersion energy, view along $\vec{a}$:

(i) *100K* ***(j)*** *275 K, 1 atm* ***(k)*** *1.71 GPa* ***(l)*** *4.08 GPa (theory)*

Dispersion energy, view along $\vec{b}$:

(m) *100K* ***(n)*** *275 K, 1 atm* ***(o)*** *1.71 GPa* ***(p)*** *4.08 GPa (theory)*

Electrostatic energy, view along $\vec{a}$:

(q) *100K* ***(r)*** *275 K, 1 atm* ***(s)*** *1.71 GPa* ***(t)*** *4.08 GPa (theory)*

Electrostatic energy, view along $\vec{b}$:

(u) *100K* ***(v)*** *275 K, 1 atm* ***(w)*** *1.71 GPa* ***(x)*** *4.08 GPa (theory)*

Figure S10.1: *Intermolecular interactions networks in the 2°AP-β structure under various temperature and applied pressure conditions, visualized using Energy Frameworks. The tubes show the location and energy of a given intermolecular interaction - the thicker the tube, the stronger the interaction. Yellow tubes describe the positive energy value of a given interaction.*

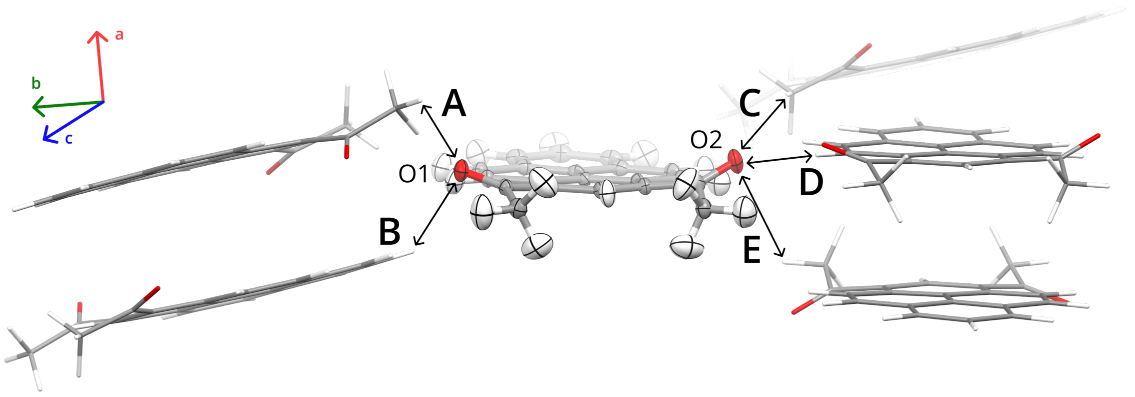

Figure S10.2: *Scheme of C-H...O interactions in the structure of 2°AP-β with symbols used in Tables below.*

Table S10.2: *Individual components of the energy of CH...O interactions A and the distances between the molecules creating them. Molecules forming this interaction are connected through the -x, y+1/2, -z+1/2 symmetry operation.*

Pressure [GPa]	Distance [Å]	Energy [kJ/mol] electrostatic	polarization	dispersion	repulsive	total
Experimental structures						
1 atm, 100 K	9.99	-14.30	-3.7	-17.4	16.9	-22.5
1 atm, 275 K	10.04	-11.70	-3.1	-16.2	13.4	-20.5
0.85	9.83	-17.80	-4.4	-20.3	25.2	-24.1
1.14	9.78	-18.30	-4.7	-21.0	27.8	-23.9
1.71	9.71	-23.10	-5.4	-22.1	31.8	-28.0
Theoretical structures						
0.06	10.25	-9.50	-1.8	-18.3	21.4	-14.0
0.30	10.25	-17.90	-2.1	-20.0	51.2	-6.2
0.56	10.14	-25.50	-2.6	-21.9	78.1	0.3
0.84	10.01	-36.50	-3.5	-24.2	117.7	10.4
1.14	9.94	-38.60	-3.7	-24.7	125.0	12.1
1.81	9.83	-60.80	-5.9	-27.0	206.3	35.3
2.30	9.73	-73.80	-7.6	-28.7	254.7	48.6
2.85	9.65	-90.40	-10.2	-30.2	316.4	66.0
3.44	9.57	-111.00	-13.9	-32.0	393.6	87.5
4.08	9.49	-138.00	-21.2	-33.9	495.8	115.2

Table S10.3: *Individual components of the energy of CH...O interactions B and the distances between the molecules creating them. Molecules forming this interaction are connected through the -x, y+1/2, -z+1/2 symmetry operation.*

Pressure [GPa]	Distance [Å]	Energy [kJ/mol] electrostatic	polarization	dispersion	repulsive	total
Experimental structures						
1 atm, 100 K	10.96	-2.4	-1.4	-12.5	11.8	-7.1
1 atm, 275 K	10.99	-1.6	-1.2	-11.3	8.9	-6.9
0.85	10.81	-2.9	-1.4	-14.7	16.3	-6.8
1.14	10.77	-3.1	-1.5	-15.5	18.2	-6.7
1.71	10.71	-3.7	-1.5	-16.1	20.5	-6.3
Theoretical structures						
0.06	11.08	-6.7	-2.7	-9.2	18.4	-5.8
0.30	11.09	-8.3	-3.1	-9.5	24.2	-4.3
0.56	11.07	-8.6	-3.2	-9.6	25.5	-4.1
0.84	11.05	-9.4	-3.3	-9.7	27.7	-3.7
1.14	10.96	-10.2	-3.5	-10.1	30.2	-3.4
1.81	10.87	-14.6	-4.3	-10.9	45.5	0.0
2.30	10.79	-18.0	-4.8	-11.5	56.8	2.4
2.85	10.71	-22.5	-5.4	-12.3	71.1	5.5
3.44	10.64	-28.4	-6.1	-13.1	89.7	9.5
4.08	10.57	-37.5	-6.9	-14.0	117.7	15.7

Table S10.4: *Individual components of the energy of CH...O interactions C and the distances between the molecules creating them. Molecules forming this interaction are connected through the x, -y+1/2, z+1/2 symmetry operation.*

Pressure [GPa]	Distance [Å]	Energy [kJ/mol] electrostatic	polarization	dispersion	repulsive	total
Experimental structures						
1 atm, 100 K	9.96	-1.4	-1.6	-17.6	10.1	-11.8
1 atm, 275 K	10.04	-0.9	-1.4	-14.9	6.7	-10.8
0.85	9.81	-2.5	-1.9	-20.7	14.6	-12.9
1.14	9.76	-3.3	-1.8	-21.8	17.1	-13.3
1.71	9.68	-4.2	-2.3	-25.3	24.5	-13.1
Theoretical structures						
0.06	10.11	1.1	-1.0	-15.0	11.3	-5.7
0.30	10.09	0.7	-1.1	-15.7	13.1	-5.7
0.56	10.03	0.0	-1.2	-17.0	17.0	-5.3
0.84	10.00	-0.6	-1.3	-17.8	20.2	-4.6
1.14	9.90	-1.1	-1.3	-18.7	22.3	-4.6
1.81	9.81	-3.9	-1.7	-21.4	35.4	-2.1
2.30	9.73	-5.7	-1.8	-22.8	42.7	-0.7
2.85	9.66	-7.8	-1.9	-23.7	51.0	1.2
3.44	9.59	-9.9	-2.1	-24.5	59.6	3.5
4.08	9.55	-11.3	-2.2	-25.2	64.8	4.5

Table S10.5: *Individual components of the energy of CH...O interactions D and the distances between the molecules creating them. Molecules forming this interaction are connected through the x, y, z symmetry operation.*

Pressure [GPa]	Distance [Å]	Energy [kJ/mol] electrostatic	polarization	dispersion	repulsive	total
		Experimental structures				
1 atm, 100 K	10.91	-6.4	-1.5	-9.6	6.7	-12.0
1 atm, 275 K	11.16	-4.8	-1.1	-7.8	4.4	-9.9
0.85	10.74	-7.8	-1.9	-11.4	10.2	-13.2
1.14	10.66	-8.5	-2.0	-12.2	12.2	-13.6
1.71	10.55	-9.2	-2.2	-13.5	16.7	-12.8
		Theoretical structures				
0.06	11.14	-6.0	-1.4	-8.8	6.5	-11.0
0.30	10.78	-7.8	-1.9	-10.9	9.3	-13.4
0.56	10.70	-8.5	-2.1	-11.7	10.9	-14.0
0.84	10.64	-9.1	-2.3	-12.5	12.6	-14.4
1.14	10.62	-9.3	-2.3	-12.7	13.2	-14.4
1.81	10.44	-12.1	-3.0	-15.2	20.6	-15.5
2.30	10.40	-13.2	-3.2	-16.0	23.6	-15.6
2.85	10.33	-14.8	-3.5	-17.0	28.3	-15.5
3.44	10.26	-16.7	-3.9	-18.0	33.9	-15.3
4.08	10.20	-18.6	-4.2	-18.8	39.1	-15.0

Table S10.6: *Individual components of the energy of CH...O interactions E and the distances between the molecules creating them. Molecules forming this interaction are connected through the -x, -y, -z symmetry operation.*

Pressure [GPa]	Distance [Å]	Energy [kJ/mol] electrostatic	polarization	dispersion	repulsive	total
		Experimental structures				
1 atm, 100 K	10.60	-4.7	-1.1	-13.1	7.2	-12.8
1 atm, 275 K	10.91	-3.6	-0.9	-10.6	4.7	-10.8
0.85	10.41	-4.8	-1.2	-15.1	9.1	-13.5
1.14	10.32	-6.1	-1.4	-16.1	9.7	-15.5
1.71	10.19	-7.0	-1.6	-17.8	11.5	-16.9
		Theoretical structures				
0.06	10.84	-5.1	-1.2	-10.7	5.4	-12.3
0.30	10.44	-5.8	-1.5	-13.1	7.3	-14.1
0.56	10.31	-6.1	-1.6	-14.4	8.4	-14.9
0.84	10.22	-6.2	-1.6	-15.3	9.3	-15.4
1.14	10.19	-6.6	-1.6	-16.1	10.6	-15.7
1.81	9.99	-8.1	-1.9	-20.0	16.0	-17.4
2.30	9.95	-8.9	-2.0	-21.4	19.1	-17.7
2.85	9.87	-10.1	-2.1	-23.5	23.4	-18.2
3.44	9.80	-11.7	-2.3	-25.8	29.0	-18.6
4.08	9.75	-12.3	-2.3	-26.7	31.4	-18.6

Chapter S2

Bibliography

[Cliffe and Goodwin, 2012] Cliffe, M. J. and Goodwin, A. L. (2012). PASCal: a principal axis strain calculator for thermal expansion and compressibility determination. *Journal of Applied Crystallography*, 45(6):1321–1329.

[Dolomanov et al., 2009] Dolomanov, O. V., Bourhis, L. J., Gildea, R. J., Howard, J. A. K., and Puschmann, H. (2009). *OLEX2* : a complete structure solution, refinement and analysis program. *Journal of Applied Crystallography*, 42(2):339–341.

[Dovesi et al., 2018] Dovesi, R., Erba, A., Orlando, R., Zicovich-Wilson, C. M., Civalleri, B., Maschio, L., Rérat, M., Casassa, S., Baima, J., Salustro, S., and Kirtman, B. (2018). Quantum-mechanical condensed matter simulations with CRYSTAL. *WIREs Computational Molecular Science*, 8(4):e1360.

[Groom et al., 2016] Groom, C. R., Bruno, I. J., Lightfoot, M. P., and Ward, S. C. (2016). The Cambridge Structural Database. *Acta Crystallographica Section B*, 72(2):171–179.

[Macrae et al., 2008] Macrae, C. F., Bruno, I. J., Chisholm, J. A., Edgington, P. R., McCabe, P., Pidcock, E., Rodriguez-Monge, L., Taylor, R., van de Streek, J., and Wood, P. A. (2008). *Mercury CSD 2.0* – new features for the visualization and investigation of crystal structures. 41(2):466–470.

[Meindl and Henn, 2008] Meindl, K. and Henn, J. (2008). Foundations of residual-density analysis. *Acta Crystallographica Section A*, 64(3):404–418.

[Rajagopal et al., 2014] Rajagopal, S. K., Philip, A. M., Nagarajan, K., and Hariharan, M. (2014). Progressive acylation of pyrene engineers solid state packing and colour via c–h$\cdots$h–c, c–h$\cdots$o and π–π interactions. *Chem. Commun.*, 50(63):8644–8647.

[Rigaku-Oxford-Diffraction, 2019] Rigaku-Oxford-Diffraction (2019). CrysAlisPro software system. version 1.171.38.46, Rigaku Oxford Diffraction.

[Tchoń and Makal, 2021] Tchoń, D. and Makal, A. (2021). Maximizing completeness in single-crystal high-pressure diffraction experiments: phase transitions in 2AP. *IUCrJ*, 8(6):1006–1017.
